# Protecting Firefighters from Carcinogenic Exposure: Emerging Tools for PAH Detection and Decontamination

**DOI:** 10.3390/bios15080547

**Published:** 2025-08-20

**Authors:** Morteza Ghafar-Zadeh, Azadeh Amrollahi Biyouki, Negar Heidari, Niloufar Delfan, Parviz Norouzi, Sebastian Magierowski, Ebrahim Ghafar-Zadeh

**Affiliations:** 1 Chips & Digital Biology Laboratory, Department of EECS, Lassonde School of Engineering, York University, 4700 Keele Street, Toronto, ON M3J 1P3, Canada; magiero@eecs.yorku.ca; 2 Biologically Inspired Sensors and Actuators Laboratory (BioSA), Department of EECS, Lassonde School of Engineering, York University, 4700 Keele Street, Toronto, ON M3J 1P3, Canada; aab@yorku.ca (A.A.B.); heidari.negar@ut.ac.ir (N.H.); ndelfan@yorku.ca (N.D.); pnorouzi@yorku.ca (P.N.); 3 Center of Excellence in Electrochemistry, Faculty of Chemistry, University of Tehran, Tehran 1439957131, Iran

**Keywords:** polycyclic aromatic hydrocarbons (PAHs), firefighter health and safety, carcinogenic exposure, portable sensing technologies, PAH decontamination, field-deployable sensors

## Abstract

Polycyclic aromatic hydrocarbons (PAHs) are increasingly recognized as a major contributor to the occupational cancer risk among firefighters. In response, the National Fire Protection Association (NFPA) and other regulatory bodies have recommended rigorous decontamination protocols to minimize PAH exposure. Despite these efforts, a critical gap persists: the absence of real-time, field-deployable devices capable of detecting these invisible and toxic compounds during firefighting operations or within fire stations. Additionally, the lack of effective and optimized methods for the removal of these hazardous substances from the immediate environments of firefighters continues to pose a serious occupational health challenge. Although numerous studies have investigated PAH detection in environmental contexts, current technologies are still largely confined to laboratory settings and are unsuitable for field use. This review critically examines recent advances in PAH decontamination strategies for firefighting and explores alternative sensing solutions. We evaluate both conventional analytical methods, such as gas chromatography, high-performance liquid chromatography, and mass spectrometry, and emerging portable PAH detection technologies. By highlighting the limitations of existing systems and presenting novel sensing approaches, this paper aims to catalyze innovation in sensor development. Our ultimate goal is to inspire the creation of robust, field-deployable tools that enhance decontamination practices and significantly improve the health and safety of firefighters by reducing their long-term risks of cancer.

## 1. Introduction

Firefighters worldwide demonstrate unwavering dedication to public safety, frequently to the detriment of their own health. Occupational cancer accounts for over 86% of work-related fatalities among firefighters, with firefighters facing a 9% greater incidence of cancer and a 14% higher risk of cancer-related mortality. One of the primary contributors to this elevated cancer risk is exposure to polycyclic aromatic hydrocarbons (PAHs)—toxic compounds generated during the combustion of organic materials such as wood, plastics, and various synthetic substances [1,2,3]. PAHs, including the well-known carcinogen benzo[a]pyrene (BaP), represent a significant health hazard due to their carcinogenic and mutagenic properties. Classified as Group 1 carcinogens by the International Agency for Research on Cancer (IARC) [4], PAHs have been shown to cause oxidative stress, DNA damage, genomic instability, and hormonal disruption [5]. Once absorbed, they accumulate in tissues such as the liver and lungs, leading to lipid peroxidation, DNA degradation, and progressive cellular damage [6]. Biomonitoring studies have confirmed PAHs’ presence in the urine, blood, and breast milk of female firefighters, indicating systemic exposure [7,8,9,10,11,12]. A large-scale study by Daniels et al., involving nearly 30,000 U.S. firefighters, identified elevated risks for cancers of the respiratory, digestive, and urinary systems [13]. These findings have been further corroborated by recent surveillance data from the National Institute for Occupational Safety and Health (NIOSH) [14], which emphasize the strong link between long-term PAH exposure and cancer prevalence in this population. Together, this evidence underscores the urgent need for improved PAH detection and decontamination technologies to mitigate occupational health risks among firefighters.

**Figure 1:**
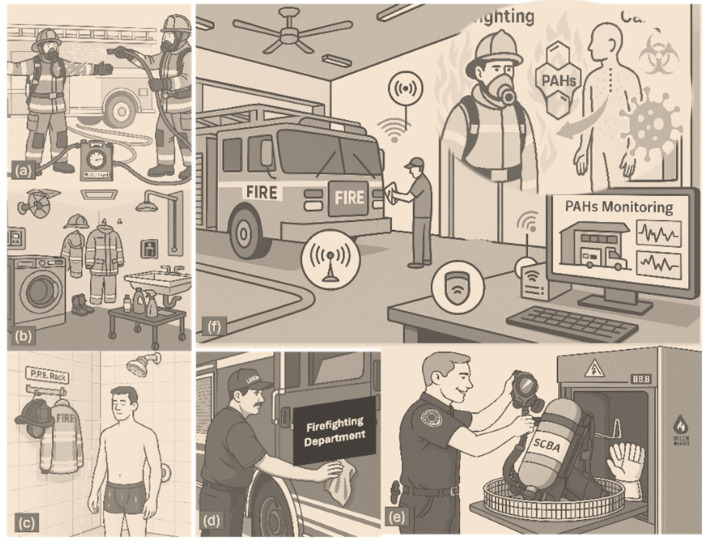
This illustration presents a holistic view of firefighter exposure to polycyclic aromatic hydrocarbons (PAHs) and the multilayered decontamination procedures implemented to mitigate the associated cancer risks. Scene (**a**) depicts on-site gross decontamination, where firefighters are sprayed down using a DECON/pak system to reduce immediate surface contamination. Scene (**b**) shows the designated gear room, highlighting organized PPE storage and laundering facilities, essential for routine cleaning. In (**c**), a firefighter showers post-response to remove dermally absorbed PAHs, emphasizing skin decontamination as a critical step. Scene (**d**) captures fire truck surface cleaning to eliminate persistent contaminants from high-contact areas. Scene (**e**) illustrates specialized SCBA washer use for the deep decontamination of respiratory equipment. Finally, (**f**) depicts the integration of digital PAH monitoring systems within the fire station, linking real-time environmental sensor data health risk models that visualize PAH absorption pathways and their connection to cancer development.

To mitigate PAH exposure, guidelines from organizations such as the National Fire Protection Association (NFPA) recommend procedures to minimize contamination during and after firefighting activities, both on-scene and upon return to the station (see Figure 1). Firefighters’ personal protective equipment (PPE) provides essential thermal protection but offers limited resistance to chemical contaminants like PAHs [8,15,16,17]. Typically composed of an outer shell, moisture barrier, and thermal liner, PPE becomes increasingly permeable as the fabric pores expand under high heat and humidity, allowing PAHs to infiltrate the gear [17,18]. These contaminants can subsequently transfer to high-contact surfaces, such as steering wheels and seats, forming persistent “hotspots” due to the chemical stability and hydrophobic nature of PAHs. Environmental conditions—such as poor air quality, elevated humidity, and high temperatures—further exacerbate the issue by promoting re-aerosolization and increasing the risk of secondary exposure [15,19,20,21].

Although self-contained breathing apparatuses (SCBAs) protect against inhalation, dermal absorption remains a critical exposure route, especially under high-heat conditions that increase skin permeability. Anatomical regions such as the neck, wrists, and forearms are particularly vulnerable. Current decontamination protocols recommend an immediate gross decontamination (“gross decon”) phase, where water or mild soap is used to rinse surface contaminants from gear. Wet decontamination has been shown to remove up to 85% of PAHs from turnout gear, while dry brushing in cold conditions removes only around 23% [21,22]. Cleansing wipes used on exposed skin (e.g., neck, face, and hands) can eliminate approximately 54% of surface PAHs [23,24,25]. To minimize off-gassing and prevent cross-contamination, contaminated gear should be stored outside of vehicle cabins whenever possible.

Post-response cleaning involves advanced laundering systems and detergents specifically formulated to dislodge PAHs from fabrics. Non-ionic and charcoal-based detergents have demonstrated high efficacy, particularly when used with pre-soaking or in higher concentrations [26,27,28]. For personal hygiene, skin-specific decontamination products are applied during post-exposure showers; however, complete PAH removal remains challenging, with effectiveness ranging from 40% to 90%, depending on the specific compound and level of contamination [26].

Despite these interventions, several critical procedural and technical questions remain unresolved. For instance, what is the optimal timing for SCBA removal—during vehicle transit or after returning to the station? Should firefighters shower in vehicle-installed systems or wait until they return to the station? The efficacy of air jets prior to vehicle entry is also unclear, as they may redistribute rather than remove surface contaminants. Additionally, there is no standardized approach for laundering protocols, including detergent selection, water temperature, cycle speed, and duration. Similarly, in-station air filtration systems require further evaluation in terms of filter types, replacement intervals, and the effects of ambient conditions such as temperature and humidity. For skin decontamination, optimal parameters for the water temperature and showerhead configuration remain undefined.

Modern fires increasingly involve synthetic materials, resulting in higher PAH concentrations at fire scenes and within fire stations. Recent studies have shown that existing decontamination methods are insufficient to fully eliminate PAHs, which are chemically stable, adhere to surfaces, and persist across both airborne and aqueous phases—complicating their detection and removal [15]. A major limitation is the absence of real-time PAH monitoring systems in fire stations, vehicles, and field environments. This gap is driven by the lack of cost-effective, field-deployable technologies capable of continuous measurement. Standard methods such as gas chromatography–mass spectrometry (GC-MS), while highly accurate, are prohibitively expensive, slow, and impractical for on-site use [29]. PAH contamination is not limited to the fireground; it can spread throughout apparatus bays, vehicle interiors, gear rooms, and even adjacent areas of the station [27]. These contaminants accumulate on gear, skin, and high-contact surfaces, posing persistent secondary exposure risks [30]. Turnout gear, in particular, can absorb and retain PAHs, which may off-gas under varying thermal or humidity conditions—sustaining the contamination cycle. This research initiative aims to address these critical gaps by assessing environmental PAH levels and evaluating the effectiveness of existing decontamination strategies and monitoring technologies. The long-term goal is to inform and elevate decontamination standards, ultimately improving firefighter safety and reducing long-term cancer risks.

This manuscript addresses the growing occupational health threat posed by polycyclic aromatic hydrocarbons (PAHs) in firefighting environments, driven by the increasing combustion of synthetic materials and the persistence of PAHs across dermal, airborne, and surface contact pathways. Despite the implementation of decontamination protocols and advancements in analytical chemistry, current methods fall short in achieving timely, field-ready PAH detection. The central aim of this work is to critically evaluate existing decontamination strategies and sensing technologies, identify unresolved challenges, and articulate a phased roadmap toward the development of integrated, real-time PAH monitoring systems suitable for firefighting operations. Section 1 provides an overview of PAH toxicology and exposure dynamics in fire service settings. Section 2 explores the physicochemical complexities that hinder effective detection and remediation. Section 3 analyzes current decontamination procedures and their limitations. Section 4 reviews the capabilities and constraints of both laboratory-based and portable sensing platforms. Finally, Section 5 integrates these insights to propose technological directions for future biosensor development in this high-risk occupational context.

## 2. PAHs: Chemical Properties and Environmental Behavior

Polycyclic aromatic hydrocarbons (PAHs) are organic compounds composed exclusively of carbon and hydrogen atoms, consisting of two or more fused benzene rings [23]. Their extended π-electron systems confer resonance stabilization, resulting in high thermodynamic stability and chemical inertness [23,24]. This planar and rigid geometry enables strong π–π stacking interactions, contributing to their persistence in environmental matrices such as soil, sediments, and airborne particulate matter [25].

The structural configuration of PAHs—whether linear, angular, or clustered—directly influences their hydrophobicity, volatility, and environmental mobility, which in turn affect their bioavailability, metabolic activation, and toxicological properties [23,26].

Physicochemically, PAHs exhibit low vapor pressures, high melting and boiling points, and minimal aqueous solubility, consistent with their non-polar nature and rigid molecular frameworks [27,28]. These properties favor their partitioning into organic phases and adsorption onto particulates. Their environmental distribution depends on the molecular weight and ring number: low-molecular-weight PAHs (two to three rings) are relatively volatile and exist in the gas phase, whereas high-molecular-weight PAHs (≥five rings) preferentially bind to airborne particles, decreasing mobility but increasing persistence [31].

PAHs are primarily released through anthropogenic processes, particularly the incomplete combustion of fossil fuels and organic matter in industrial operations, transportation, residential heating, and waste incineration. Due to their lipophilicity and stability, PAHs accumulate in lipid-rich tissues such as the liver and adipose tissue following inhalation, ingestion, or dermal absorption [31]. Carcinogenic compounds such as benzo[a]pyrene (BaP) undergo metabolic activation by cytochrome P450 enzymes to form reactive intermediates, including diol epoxides, which generate DNA adducts and interfere with replication and repair pathways—initiating mutagenesis and carcinogenesis [6,25].

Concurrently, PAH metabolism generates reactive oxygen species (ROS), leading to oxidative stress, lipid peroxidation, protein carbonylation, and further DNA damage [8]. These mechanisms contribute to a range of adverse health outcomes, including lung, skin, and breast cancers, developmental toxicity, and epigenetic alterations associated with aging [32].

**Figure 2:**
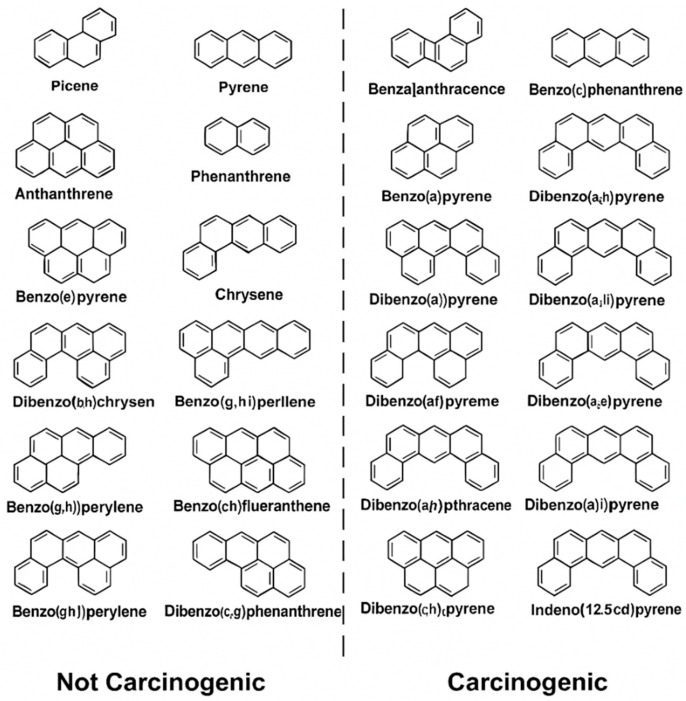
Structures of common polycyclic aromatic hydrocarbons (PAHs).

Given the persistence and biological activity of PAHs—especially in high-risk professions such as firefighting—there is a critical need for sensitive, real-time monitoring systems to assess exposure. Figure 2 presents the structures of representative carcinogenic PAHs, whose toxic potential increases with the number of fused aromatic rings due to enhanced lipophilicity, environmental stability, and DNA-binding capacity [33,34].

Higher-molecular-weight PAHs (HMW-PAHs), particularly those with four or more fused aromatic rings, are strongly associated with carcinogenicity, environmental persistence, and resistance to metabolic clearance. Compounds such as dibenzo[a,l]pyrene, benz[a]anthracene, and chrysene—alongside the frequently studied benzo[a]pyrene (BaP)—are classified by the IARC as Group 1 or 2 carcinogens due to established links with lung, bladder, and skin cancers [35]. These PAHs undergo metabolic activation via cytochrome P450 enzymes to form electrophilic intermediates capable of binding DNA and inducing genomic instability [36]. In addition to genotoxicity, long-term PAH exposure has been implicated in developmental toxicity, immune suppression, and endocrine disruption, as highlighted by the U.S. EPA and National Toxicology Program [37,38]. The diversity and potency of these compounds underscore the urgent need for occupational exposure assessment tools that are not only highly sensitive but also capable of multi-analyte detection. This is especially critical in professions such as firefighting and chemical processing, where real-time monitoring and protective strategies remain limited.

## 3. Firefighters and PAHs

Firefighting is a high-risk occupation characterized by chronic exposure to a wide array of combustion-derived toxicants, including polycyclic aromatic hydrocarbons (PAHs), particulates, asbestos, diesel exhaust, flame retardants, and per- and polyfluoroalkyl substances (PFAS). Among these, PAHs are particularly concerning due to their high environmental persistence, capacity for dermal and respiratory absorption, and well-established carcinogenicity. Firefighters encounter PAHs through multiple exposure routes during active fire suppression, overhaul phases, and the subsequent handling of contaminated gear [39,40].

### 3.1. Characteristics of Personal Protective Equipment of Firefighters

**Figure 3:**
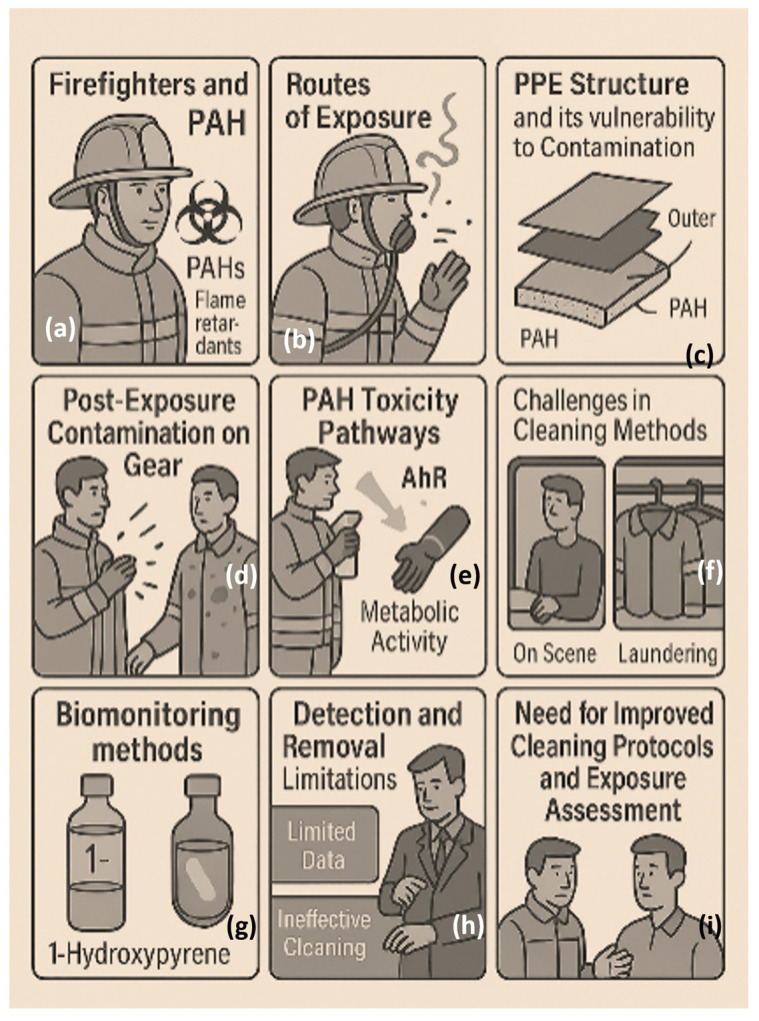
Firefighting PAH challenges: (**a**) *Firefighters and PAHs:* Firefighters face elevated cancer risks due to chronic exposure to PAHs and other toxicants during fire suppression. (**b**) *Routes of Exposure:* PAHs can enter the body through inhalation, dermal absorption, and ingestion, especially during active firefighting and overhaul. (**c**) *PPE Structure and Vulnerability:* Multi-layered PPE is designed for thermal protection but can accumulate PAHs, especially in outer and thermal layers. (**d**) *Post-Exposure Contamination:* PAHs persist on gear and skin post-fire, leading to secondary exposure if not properly decontaminated. (**e**) *PAH Toxicity Pathways:* PAHs bind to the aryl hydrocarbon receptor (AhR), triggering metabolic activation and toxic responses, including DNA damage. (**f**) *Challenges in Cleaning Methods:* On-scene gross decon and laundering protocols vary in effectiveness; water alone is often insufficient for PAH removal. (**g**) *Biomonitoring Methods:* Urinary 1-hydroxypyrene is used as a biomarker to assess internal PAH exposure among firefighters. (**h**) *Detection and Removal Limitations:* Limited toxicological data and inadequate cleaning protocols hinder effective monitoring and risk reduction. (**i**) *Need for Improved Protocols:* There is a pressing need to develop standardized cleaning procedures and exposure assessment tools to reduce firefighter health risks.

Personal protective equipment (PPE) is essential in shielding firefighters from thermal exposure, flame contact, mechanical injuries, and the environmental hazards encountered during rescue operations and fire suppression (Figure 3). The standard PPE ensemble includes a multi-layered protective suit, a helmet, gloves, a balaclava, and fire-resistant boots [30]. 65tyt plays a pivotal role and comprises four key layers: an outer shell, moisture barrier, thermal barrier, and inner liner. The outer shell, typically constructed from flame-resistant fabrics such as aramids (e.g., Nomex^®^, Kevlar^®^), polyamides (e.g., Kermel^®^), polyimides (e.g., Lenzing^®^), or polybenzimidazoles (e.g., PBI), provides mechanical strength and heat resistance [30]. Its light color (e.g., golden or sand) makes contamination visible, promoting regular cleaning—an essential practice in minimizing chemical exposure. The moisture barrier, composed of microporous, waterproof, and vapor-permeable membranes (commonly PTFE, PE, or PU), prevents external moisture ingress while allowing perspiration to escape [41,42]. The thermal barrier includes flame-resistant woven or laminated non-woven fabrics, typically composed of aramid, polyester, or aramid–viscose blends, and protects against radiant heat [41]. The inner liner, either independent or integrated with the thermal barrier, is usually formed from aramid–viscose blends, fully aramid fabrics, or treated cotton materials. The thermal and flame resistance of these layers is often evaluated using the Limiting Oxygen Index (LOI), which quantifies the minimum oxygen concentration required to sustain combustion [43].

Despite this multi-layered design, current PPE has limited chemical resistance, particularly against polycyclic aromatic hydrocarbons (PAHs). These persistent, lipophilic compounds can penetrate garment seams and fabric microstructures during high-temperature exposure. PAHs such as benzo[a]pyrene and dibenzo[a,l]pyrene can adsorb to inner layers and persist even after standard laundering due to strong fiber–PAH interactions and poor aqueous solubility [44]. This contamination leads to prolonged dermal and inhalation exposure through off-gassing—a process in which absorbed chemicals are re-emitted from fabrics under post-incident conditions, especially when gear is stored in poorly ventilated environments. Wilkinson et al. demonstrated that residual PAHs could remain in gear long after deployment, particularly when decontamination, ventilation, or temperature control are insufficient [45]. Moreover, fabric porosity, seam permeability, and the thermal degradation of barriers contribute to PPE’s inability to fully block PAH ingress. Consequently, there is a pressing need to develop advanced protective materials featuring higher chemical permeation resistance, sealed seams and joints, embedded passive PAH dosimeters, and validated decontamination protocols optimized for PAH desorption. These advancements are critical in reducing the long-term carcinogenic risks faced by firefighters.

### 3.2. Assessment of the Toxicity of Firefighter Exposures

Polycyclic aromatic hydrocarbons (PAHs) remain among the most toxic constituents of combustion byproducts encountered by firefighters. Numerous studies have quantified their concentrations in smoke from both live fire incidents and training exercises, as well as on contaminated skin and gear through swab analysis [9,46,47,48,49,50]. While self-contained breathing apparatuses (SCBAs) offer effective protection against inhalation, dermal exposure has emerged as a dominant route of uptake—particularly in anatomical interface zones such as the neck, wrists, and jawline. Post-incident biomonitoring has consistently detected elevated levels of PAH metabolites in the urine of firefighters, confirming systemic absorption even when respiratory protection is in use [50,51,52]. However, current analytical approaches often rely on targeted mass spectrometry, which, despite its sensitivity, is limited to predefined compounds. This narrow scope may underestimate the total toxic load, as firefighting environments typically involve exposure to complex, multi-component mixtures of PAHs and other combustion-derived contaminants.

Mechanistically, PAH toxicity is largely mediated by the activation of the aryl hydrocarbon receptor (AhR), a ligand-dependent transcription factor that regulates xenobiotic metabolism. Upon binding, PAHs undergo metabolic activation via cytochrome P450 enzymes, yielding reactive intermediates capable of forming DNA adducts and inducing oxidative stress, inflammation, and mutagenesis [53,54]. Elevated ambient temperatures during firefighting not only increase PAHs’ volatility but also enhance percutaneous absorption by disrupting the skin barrier function [54,55]. These factors together amplify the internal dose, particularly during extended operations or when decontamination is delayed. Given the variation in PAH toxicity, exposure duration, and individual susceptibility, there is a growing need for untargeted analytical tools that can capture the full spectrum of exposure, as well as real-time biosensing platforms capable of tracking dermal uptake dynamics. Such innovations would complement existing protective strategies and support more comprehensive health risk assessments for firefighters.

### 3.3. Challenges in PAH Removal from Firefighting Gear

Firefighting activities expose personnel to high levels of PAHs, which can adsorb onto the outer shells of protective gear and permeate into the inner layers under elevated temperatures. To address this, the National Fire Protection Association (NFPA) recommends two decontamination levels: on-scene routine cleaning and machine-based advanced laundering [56]. Routine methods—such as soap-and-water scrubbing—can remove up to 85% of surface PAHs, whereas dry brushing and air blowing achieve significantly lower efficiencies (24% and 0.5%, respectively) [57]. However, due to the hydrophobicity and structural persistence of PAHs, water-only cleaning remains largely ineffective [56]. Advanced laundering using commercial detergents and surfactants improves the solubilization of hydrophobic compounds, yet PAHs embedded in textile pores or laminated barrier layers often remain unrecovered [58,59,60]. For example, studies by Sánchez-Alvarado and Girase reported minimal changes in PAH, PBDE, and OPFR levels before and after laundering [61,62]. The effectiveness of available fire service-specific cleaning agents, such as Citrosqueeze^®^, Turnout Gear Wash, and Doff ‘n DECON™, is typically based on manufacturer guidelines rather than independent PAH-specific validation. Moreover, NFPA 1851 lacks standardized recommendations regarding the detergent composition, concentration, or laundering conditions, leading to inconsistent practices across departments [63,64,65]. The degree of contamination varies with the PPE design, exposure severity, and firefighting role—especially for interior attack personnel. High-molecular-weight PAHs, in particular, show poor water solubility and bind tightly to fabrics, making removal difficult. Repeated laundering can even degrade fire-retardant coatings, reducing PPE’s lifespan and performance. Furthermore, the absence of integrated detection tools makes it difficult to assess residual contamination or cleaning efficacy.

To better address these limitations, emerging strategies such as solvent-assisted extraction, ozone oxidation, and nanomaterial-enhanced detergents are being explored. These approaches aim to increase the removal efficiency, reduce toxic residues, and prolong gear usability. Figure 3 illustrates key PAH absorption routes, retention zones, and post-exposure cleaning challenges.

### 3.4. PAH Exposure Assessment

The most common method of assessing PAH exposure involves analyzing tissues, blood, or urine for metabolites such as 1-hydroxypyrene, a biomarker derived from pyrene and widely used as a biological exposure index (BEI) [66,67,68,69]. The ACGIH recommends its measurement in end-of-shift urine samples to identify workplaces with elevated PAH exposure. Data from the NHANES survey reported a geometric mean of 74.2 ng/g creatinine for 1-hydroxypyrene in the U.S. population aged six and older. While these values provide reference ranges, they do not establish health risk thresholds, and individual interpretation remains limited by variability, confounding factors, and the lack of specificity at low exposure levels [69,70,71].

## 4. PAH Monitoring

The choice of sensing technology for PAH detection is influenced by the application’s specific requirements, including sensitivity, selectivity, and the nature of the sample matrix. Fluorescence and SERS sensors provide excellent sensitivity and specificity and are suitable for laboratory settings and detailed analysis [72]. Conversely, electrochemical sensors offer significant advantages for portable, on-site detection due to their ease of use, rapid response, and ability to operate in varied environmental conditions. These attributes underscore the importance of electrochemical sensing as a versatile and practical method for environmental monitoring and public health protection, facilitating real-time analysis and immediate decision making. With regular sample preparation from firefighters (e.g., skin, clothes, building HVAC system, shower, urine), novel electrochemical sensing technologies can be developed to analyze PAHs collected. Additionally, integrating these sensors into firefighting gear to continuously monitor PAHs on the skin and clothing can provide immediate feedback and reduce long-term health risks by enabling quicker interventions. This approach enhances firefighter safety and health outcomes and provides valuable data for the development of better protective strategies and protocols. Based on the literature, the samples should be in a liquid format or be solved in liquids for detection. Therefore, all samples collected from firefighters can be analyzed with electrochemical and charge-sensitive sensors like FET sensors for the direct detection of airborne particles. Traditional analytical techniques such as gas chromatography (GC), high-performance liquid chromatography (HPLC), mass spectrometry (MS), optical fluorescence, and surface-enhanced Raman scattering (SERS) are considered gold-standard methods due to their high precision and sensitivity [56,73,74,75,76,77,78]. However, these techniques are often constrained to laboratory use because of complex sample preparation, calibration requirements, and limited field portability.

Recent research has therefore shifted toward developing field-deployable solutions that can provide real-time PAH detection. One promising approach involves the use of electrochemical and field-effect transistor (FET) sensors integrated with nanomaterials such as graphene and carbon nanotubes, which enhance sensor performance by increasing the charge sensitivity in the presence of PAHs [79,80]. These systems allow the in situ monitoring of the total PAH concentration (TPC) without requiring laboratory infrastructure. However, sensor durability remains a challenge due to the difficulty of desorbing PAHs from the sensing surface, necessitating innovations in thermal/electrochemical regeneration and the development of self-cleaning or hybrid materials [39]. Artificial intelligence (AI) is increasingly being integrated into sensor platforms to enhance specificity, automate calibration, and correct signal drift in high-risk environments. This integration has the potential to improve accuracy and reduce the need for manual adjustments [81,82].

**Figure 4:**
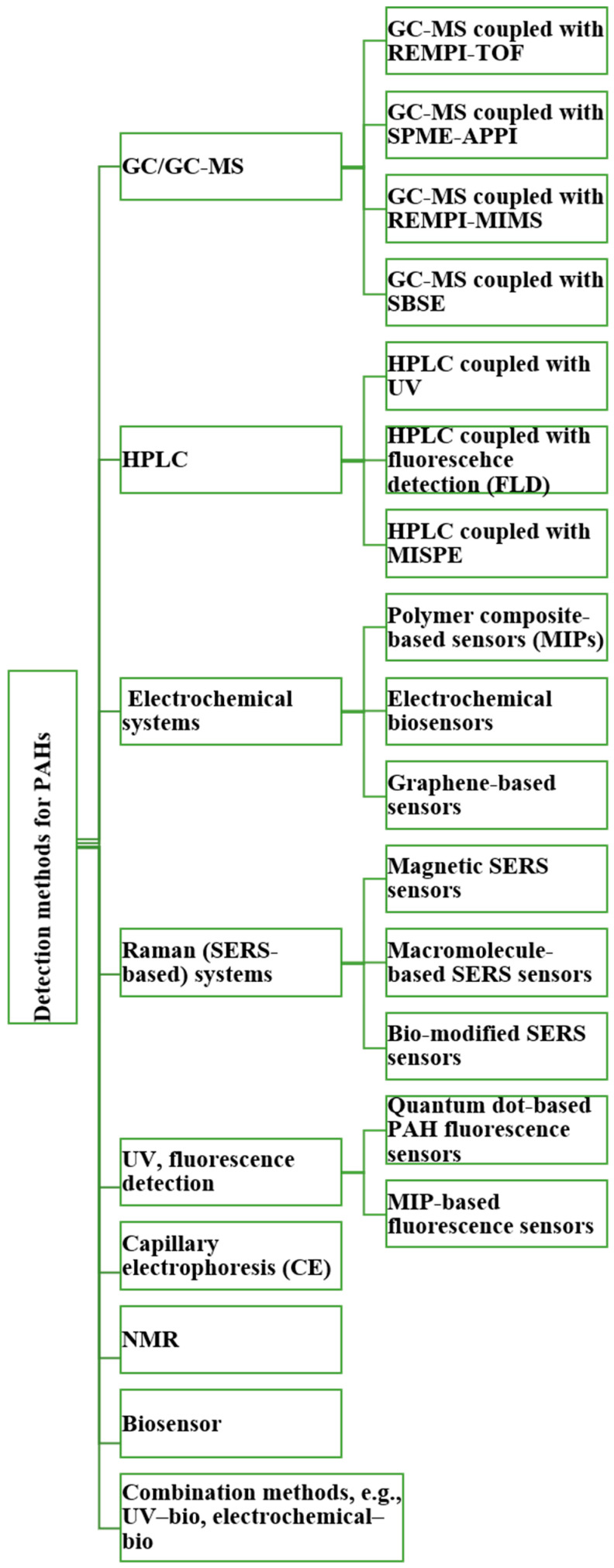
Brief overview of analytical techniques for detection of PAHs.

Meanwhile, broader analytical strategies for PAH detection have evolved to include complementary techniques. HPLC and GC remain widely used for routine analysis due to their high separation efficiency and reproducibility, while emerging methods such as capillary electrophoresis (CE), NMR spectroscopy, electrochemical sensors, nanopore systems, and molecularly imprinted polymers (MIPs) are expanding the detection capabilities. Combined approaches—for example, coupling HPLC with fluorescence detection and GC-MS with spectral libraries—enable the detailed characterization of complex PAH, PASH, and PANH mixtures. Together, these advancements highlight the growing emphasis on multi-modal, real-time, and field-compatible PAH sensing systems that are essential for protecting human health in environments such as firefighting. Overall, this section outlines key traditional and hybrid analytical approaches used in PAH research and serves as an introduction to the techniques described in greater detail in the following section (Figure 4).

### 4.1. Gas Chromatography–Mass Spectrometry (GC-MS)

**Figure 5:**
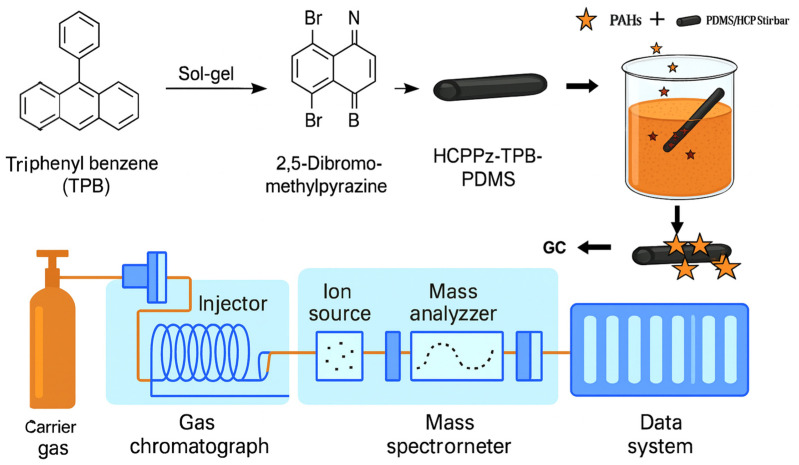
Schematic of PDMS/HCPPz-TPB stir bar preparation. A hyper-crosslinked porous polymer (HCPPz-TPB) was synthesized via the Friedel–Crafts reaction using triphenylbenzene as the monomer and 2,5-dibromomethylpyrazine as the crosslinker. The resulting polymer was integrated with polydimethylsiloxane (PDMS) through a sol–gel process and coated onto an in-house-fabricated dumbbell-shaped stir bar for PAH detection via GC-MS.

Gas chromatography (GC) remains a foundational technique for PAH analysis due to its high sensitivity, selectivity, and operational simplicity (Figure 5). Although GC struggles to separate PAHs with similar boiling points and vapor pressures, coupling with mass spectrometry (GC-MS) enables the precise identification and quantification of volatile and semi-volatile organic compounds. GC-MS is particularly valued for its molecular specificity, but challenges such as resolving structural isomers, managing matrix complexity, and high operational costs limit its accessibility in resource-constrained settings.

Given that PAHs typically occur at trace levels in complex environmental matrices, analytical tools must offer high sensitivity and robustness against interference. Mass spectrometry (MS) addresses this need through precise molecular weight determination and remains indispensable in analyzing PAHs across various media, including air, water, sediments, and biological tissues [68,69,83,84].

To enhance the analytical performance, recent developments have introduced hybrid MS platforms integrating novel extraction and ionization techniques. For example, solid-phase microextraction–atmospheric pressure photoionization MS (SPME-APPI-MS) and resonance-enhanced multiphoton ionization–time-of-flight MS (REMPI-TOFMS) improve the real-time detection capabilities, particularly in combustion and atmospheric samples. However, REMPI’s application in aqueous environments is constrained by matrix complexity and the typically low concentrations of PAHs.

Membrane inlet mass spectrometry (MIMS) provides a viable alternative for aqueous PAH analysis by enabling the selective pervaporation of analytes into MS vacuum chambers. This reduces the sample preparation burden and minimizes matrix effects. To address membrane durability issues in ion sources, external probe designs with transfer capillaries have been implemented, lowering rupture risks. The integration of REMPI with external-probe MIMS (eMIMS) offers a promising solution for the real-time, direct monitoring of aqueous PAHs by combining REMPI’s ionization specificity with MIMS’s sample introduction efficiency [85,86,87,88].

In a comparative study, Gehm et al. [86] evaluated REMPI-eMIMS and GC-MS for the detection of PAHs—specifically naphthalene, acenaphthene, fluorene, and phenanthrene—in spiked water samples. GC-MS employed liquid–liquid extraction with hexane and analysis via an Agilent 7010A Triple Quadrupole system. REMPI-eMIMS measurements, initially conducted four days after sample preparation, aligned well with GC-MS for naphthalene but showed deviations for phenanthrene. However, results obtained from freshly prepared samples demonstrated close agreement between both methods, indicating that storage-related interactions, particularly with container walls, had influenced earlier measurements.

Table 1 presents the comparative results for naphthalene and phenanthrene. While both methods produced consistent data for naphthalene, discrepancies in phenanthrene quantification were resolved upon the immediate analysis of fresh samples, confirming that the storage conditions can significantly affect REMPI-eMIMS’s measurement accuracy.

In a related development, the stir bar sorptive extraction (SBSE) technique has been effectively combined with GC-MS for PAH quantification in edible oil matrices. This method improves efficiency by reducing pre-treatment steps, enhancing reproducibility, and lowering solvent consumption. As a result, SBSE/GC-MS offers a robust, environmentally friendly, and cost-effective solution for routine PAH monitoring in food safety and environmental applications [87,88,89,90].

The stir bar sorptive extraction (SBSE) technique has been effectively combined with gas chromatography–mass spectrometry (GC-MS) for the quantification of 15 polycyclic aromatic hydrocarbons (PAHs) in edible oil samples. Key extraction parameters—including the stirring rate, extraction time, temperature, desorption solvent, and desorption duration—were systematically optimized to enhance the efficiency. Under these optimized conditions, the method achieved recoveries ranging from 83.14% to 128.01%, with relative standard deviations (RSDs) below 13.47%, indicating excellent precision. Limits of detection (LODs) ranged from 0.04 to 0.28 ng/g in real edible oil matrices [89,90,91].

In a separate study, Wicker et al. [92] developed and validated the first online supercritical fluid extraction coupled with supercritical fluid chromatography–mass spectrometry (SFE-SFC-MS) method for the quantification of PAHs in various soil types. This hybrid analytical system directly couples the extraction step with chromatographic separation, thereby minimizing sample handling, reducing the risk of contamination, and significantly shortening the analysis time. The method was tested and validated on certified reference materials such as sediment, clay, and sand. It demonstrated excellent linearity (R^2^ ≥ 0.99) for PAH concentrations ranging from 10 to 1500 ng/g. The LODs achieved were between 0.001 and 5 ng/g, while the limits of quantification (LOQs) ranged from 5 to 15 ng/g [93,94].

**Figure 6:**
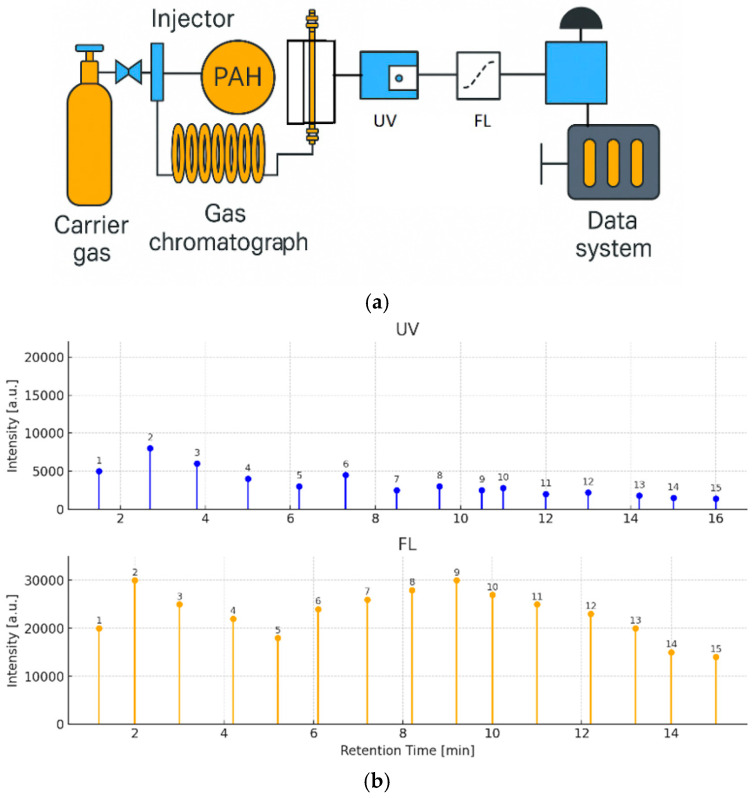
(**a**) Supercritical fluid chromatography (SFC) system schematic with UV and FL detector column: 2-ethylpyridine. (**b**) UV and FL chromatograms of PAHs (UV: 500 pg/µL, FL: 5 pg/µL, 1: Naphthalene, 2: Acenaphthene, 3: Fluorene, 4: Acenaphthylene (non-fluorescent), 5: Anthracene, 6: Phenanthrene, 7: Fluoranthene, 8: Pyrene, 9: Benzo[a]anthracene, 10: Chrysene, 11: Benzo[k]fluoranthene, 12: Benzo[b]fluoranthene, 13: Benzo[a]pyrene, 14: Dibenzo[a,h]anthracene, 15: Indeno [1,2,3-cd]pyrene, 16: Benzo[g,h,i]perylene). The measurement wavelength used for the UV/visible detector was the same as the excitation wavelength of the fluorescence detector.

This technique represents a rapid, sensitive, and environmentally friendly alternative for PAH monitoring in environmental matrices. A total of 16 priority PAHs were efficiently separated using supercritical fluid chromatography (SFC) and subsequently detected with both fluorescence (FL) and UV detection using a high-pressure flow cell (Figure 6a). The SFE-SFC-MS platform thus offers a fully integrated and automated solution for accurate and high-throughput environmental PAH analysis.

Figure 6b shows the separation of a 16-component PAH standard mixture with UV–visible detection (500 pg/µL each) and fluorescence detection (5 pg/µL each). Using the fluorescence detector, the PAHs were detected at the low detection limit of 0.17 pg to 4.6 pg, and the sensitivity ratio compared with the UV detector was approximately 20 to 400 times.

### 4.2. High-Performance Liquid Chromatography (HPLC)

High-performance liquid chromatography (HPLC) remains a widely adopted method for the qualitative and quantitative detection of PAHs, relying on analyte interactions with both stationary and mobile phases. Wang et al. [95] employed HPLC with ultraviolet (UV) and fluorescence detection to quantify 16 PAHs in water, achieving excellent linearity (R > 0.999), low detection limits (0.3–5.0 ng/L), and recoveries ranging from 67.2% to 114.1%. Zhang et al. [56] utilized HPLC coupled with fluorescence detection (HPLC-FLD) to measure PAH concentrations in barbecued meats, reporting strong linearity (R > 0.9995), recoveries between 71.1% and 98.8%, and detection limits from 0.33 to 3.30 μg/kg. In another study, Wang et al. [96] developed an online HPLC system for the detection of PAHs in river water and coal ash leachate, achieving enrichment factors between 77.6 and 678, with detection limits as low as 0.01 μg/L.

Despite these advantages, the overall performance of HPLC is constrained by the sensitivity of the detector, and it often necessitates extensive sample pre-treatment and sophisticated instrumentation and does not provide information on PAH transformation pathways.

Recent advancements in portable gas chromatography (GC), liquid chromatography (LC), and mass spectrometry (MS) technologies aim to translate laboratory-level PAH detection capabilities to field settings. However, performance trade-offs remain. Miniaturized GC-MS instruments offer portability but at the cost of resolution and sensitivity. GC-MS is best suited for detecting volatile and thermally stable PAHs, while LC systems are more appropriate for polar or thermally labile PAHs but generally lack robustness in field conditions. Portable LC devices eliminate the need for vacuum systems or carrier gases, but they are often limited to single-wavelength UV–vis detectors, reducing the specificity and increasing the risk of misidentification based on the retention time alone. While laboratory-based LC-MS systems overcome many of these issues, a fully portable LC-MS platform suitable for in-field PAH detection has not yet been commercialized [97,98].

**Figure 7:**
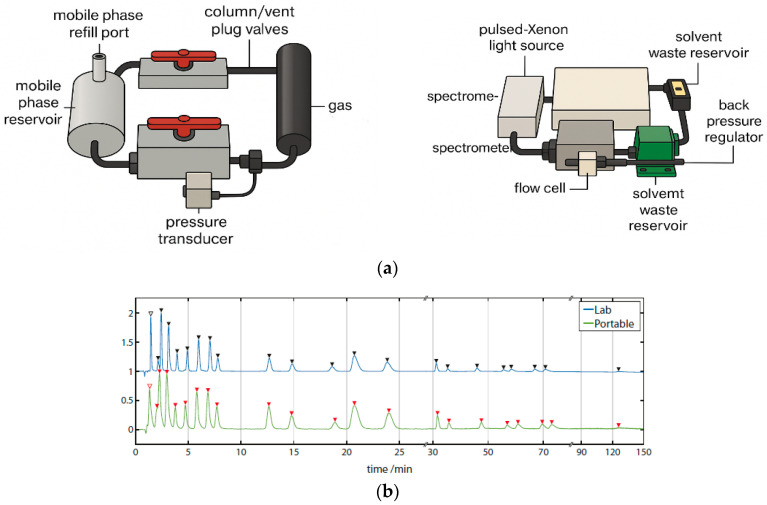
(**a**) HPLC portable chromatography system. (**b**) Chromatograms annotated with spectrally identified species. Exemplar chromatograms of the 24-component PAH separation run on the portable LC system at 230 ± 2 nm, annotated with peak identification numbers, using a Zorbax PAH and Poroshell C_18_ columns.

Using a custom-built portable HPLC system (Figure 7a), full-spectrum chromatograms were acquired for a 24-component PAH mixture. The system offered spectral coverage from 180 to 890 nm, with an optical resolution of 0.217 nm and a sampling rate of 1 Hz, extendable to 220 Hz. This represents the first documented instance of a portable HPLC capable of detecting analytes at wavelengths as low as 180 nm while simultaneously capturing full-spectrum data.

As shown in the annotated chromatograms and spectral heatmap (Figure 7b), PAHs exhibited pronounced absorption in the 230–300 nm region, with some extending beyond 400 nm. At 230 ± 2 nm, well-separated peaks with high signal-to-noise ratios (SNRs) were observed. The unique spectral absorption profiles of individual PAHs created low-congestion spectral regions, facilitating baseline separation through the disappearance of overlapping peaks.

### 4.3. Electrochemistry Methods for PAH Detection

Current methods for detecting PAHs—such as high-performance liquid chromatography (HPLC) with fluorescence detection, gas chromatography–mass spectrometry (GC-MS), and capillary electrophoresis (CE)—are highly sensitive but require complex and time-consuming sample preparation, thereby limiting their applicability in field settings. Emerging alternatives such as surface-enhanced Raman spectroscopy (SERS), fluorescence spectroscopy, and electrochemical biosensors offer rapid, in situ detection capabilities. Voltammetric biosensors, particularly for the trace-level detection of benzo[a]pyrene in water, utilize electroactive bioreceptors to enhance the analytical specificity.

Advanced sensor platforms employing covalently bonded molecular recognition layers can respond to external stimuli (e.g., light, chemicals), demonstrating potential for adaptive and selective PAH detection. Given that monocyclic and polycyclic aromatic hydrocarbons (MAHs and PAHs) pose significant global environmental threats even at nanogram-per-liter concentrations [99], there is an urgent need for rapid and portable detection strategies. Nsibande et al. [100] underscore the chronic toxicity risks associated with prolonged exposure, even at trace levels. Some recent research on metal nanoparticles is summarized in Table 2.

As presented in Table 2, electrochemical sensors utilizing advanced materials—such as MXenes (e.g., Ti_3_C_2_T_x_), metal nanoparticles (e.g., PtNPs, Ni–Co layered double hydroxides [LDHs]), and carbon nanostructures (e.g., multi-walled carbon nanotubes [MWCNTs])—coupled with techniques including differential pulse voltammetry (DPV), linear sweep voltammetry (LSV), and cyclic voltammetry (CV), demonstrate remarkable sensitivity in detecting hazardous compounds such as PAHs, phenols, and endocrine-disrupting agents. These sensors achieve detection limits in the nanomolar range and exhibit broad dynamic ranges under controlled laboratory conditions.

For fire scene applications, this class of sensors offers multiple advantages. Their electrochemical transduction mechanisms are inherently resistant to thermal and optical interference, enabling operation in environments where spectroscopic techniques may fail due to dense smoke and high levels of volatile compounds. Furthermore, electrochemical sensors are highly amenable to miniaturization, require low power consumption, and can be integrated into rugged, battery-operated devices, making them well suited for firefighter gear or portable field assessment units.

However, a critical limitation persists: most reported systems are optimized for aqueous-phase detection and lack validated real-time capabilities for the airborne analytes that dominate fireground environments, and their performance under extreme conditions remains largely unverified. Additionally, achieving high selectivity for structurally similar combustion byproducts (e.g., isomeric PAHs) remains challenging without molecularly engineered recognition layers or advanced signal deconvolution techniques.

The key translational gap, therefore, lies in advancing these high-performance electrochemical platforms from bench-scale aqueous detection to the real-time, gas-phase monitoring of pollutants in fire scenes, under harsh environmental and operational conditions. Overcoming this challenge will require the integration of pre-concentration modules, aerosol sampling mechanisms, and robust environmental shielding, while maintaining sensor fidelity and responsiveness in dynamic field settings.

Metal oxide nanoparticles such as SnO_2_, ZnO, and TiO_2_ (Table 3) have also garnered substantial attention due to their excellent physicochemical properties, biocompatibility, and catalytic activity, as noted by Murtada et al. [108]. In a related study, Subhan et al. [109] developed a ternary sensor system composed of SnO_2_–ZnO–TiO_2_, which successfully detected benzaldehyde in aqueous samples, achieving high recovery rates ranging from 99.4% to 106.1%. Some recent research on metal oxide nanoparticles is summarized in Table 3.

**Figure 8:**
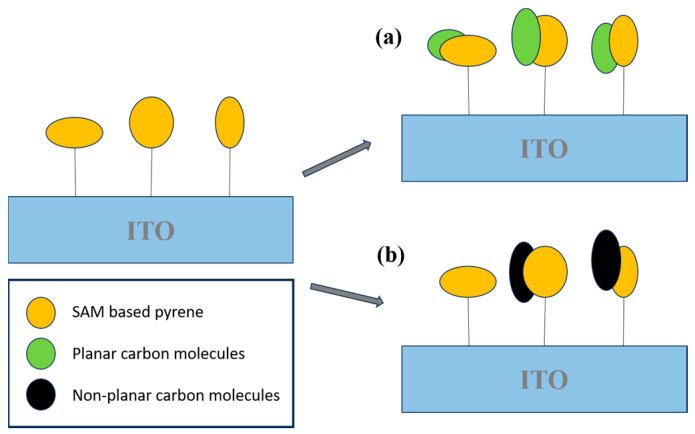
Schematic illustration of the supramolecular recognition of S3 toward (**a**) planar and (**b**) non-planar carbon molecules.

Carbon-based nanomaterials, including carbon nanotubes (CNTs), graphene (Gr), and other carbon nanostructures, are extensively employed in sensing applications due to their large surface areas, excellent electron mobility, chemical stability, and ease of surface functionalization. The integration of advanced electrochemical techniques with nanomaterial-based platforms represents a promising approach for the rapid and sensitive detection of aromatic hydrocarbons in complex environmental matrices. These sensor systems are not only scalable and portable but also provide cost-effective solutions that are ideally suited for real-time environmental monitoring and exposure risk assessment. In a notable contribution, Muñoz et al. [80] developed a highly sensitive electrochemical sensing platform for polycyclic aromatic hydrocarbons (PAHs). The system employed a hybrid material composed of a self-assembled monolayer (SAM) of an aromatic carbon compound on an indium tin oxide (ITO) substrate, specifically engineered to interact with PAH molecules. Pyrene was selected as the probe molecule due to its planar aromatic structure, which enabled strong π–π stacking interactions with PAHs. These supramolecular interactions among aromatic species facilitated the development of a highly selective and sensitive pyrene-based electrochemical sensor (Figure 8).

Carbon nanotubes (CNTs) have garnered significant attention in sensor development and nanotechnology due to their exceptional mechanical, electrical, and chemical properties. Graphene—a single layer of sp^2^-hybridized carbon atoms arranged in a two-dimensional honeycomb lattice—has emerged as a highly promising material for electrochemical sensing. Its exceptional electrical conductivity, mechanical durability, and superior electrochemical sensitivity make it a powerful candidate for the development of sensors. These properties facilitate rapid electron transfer and significant signal amplification, enabling the detection of pollutants at trace levels. To further enhance sensor performance, researchers have investigated the integration of noble metal nanoparticles—including gold (Au), silver (Ag), and palladium (Pd)—with graphene-based substrates. This combination induces a synergistic effect, significantly improving both sensitivity and selectivity. Reported nanocomposite designs include Au–Cu nanoclusters on graphene nanoribbons, Au–Pd nanoparticles on graphene nanosheets, and Au nanoparticles supported on reduced graphene oxide (rGO) combined with multi-walled carbon nanotubes (MWCNTs). These hybrid platforms have demonstrated excellent analytical performance for the detection of bisphenol A (BPA), achieving detection limits as low as 8 nM, with linear dynamic ranges spanning 0.01 to 10 µM [120].

**Figure 9:**
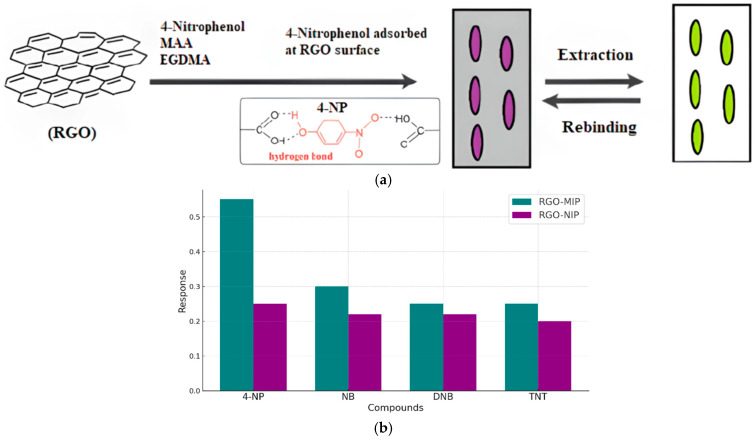
Detection of 4-NP by rGO-MIP electrochemical sensor. (**a**) Adsorption mode during detection. (**b**) Current response of 4-NP and its analogs.

Zeng et al. [121] reported the development of a novel electrochemical sensor based on a composite of reduced graphene oxide (rGO) and a molecularly imprinted polymer (MIP) for the selective and sensitive detection of 4-nitrophenol (4-NP). The rGO layer provides a large active surface area and outstanding electrical conductivity, while the MIP imparts specific recognition sites tailored to 4-NP molecules. The synergistic interaction between rGO and MIP significantly enhances the sensor’s performance, yielding improved sensitivity, selectivity, and operational stability. The fabricated sensor demonstrated a low detection limit and a wide linear response range, making it a promising tool for the environmental monitoring of 4-NP contamination (Figure 9).

Metal–organic frameworks (MOFs) are highly crystalline and porous materials composed of metal ions or clusters coordinated with organic linkers, offering exceptional surface areas, tunable pore architectures, and structural versatility, as described by Lahcen et al. [122]. These properties make MOFs excellent candidates for analyte capture and signal transduction in electrochemical sensing. In comparison, molecularly imprinted polymers (MIPs) function as synthetic recognition elements by mimicking biological receptors, forming highly selective binding cavities tailored to specific target molecules through covalent or non-covalent interactions, as reported by Lahcen et al. [122] and Yáñez-Sedeño et al. [123]. Both MOFs and MIPs have been widely explored for the development of electrochemical sensors due to their excellent selectivity and structural robustness. The structural diversity of MOFs is attributed to the flexible coordination of metal ions and organic linkers. For example,

1. Carboxylate-based linkers coordinated with divalent metal ions such as Zn^2+^, Cu^2+^, Ni^2+^, and Co^2+^ are commonly employed, although they may exhibit reduced stability in aqueous environments;
2. Alternatively, metal cluster-based frameworks with carboxylic linkers—such as Cu_4_(Me_3_CCOO)_8_(teia), AuNPs/MMPF-6(Fe), and Cu_3_(BTC)_2_(H_2_O)—offer improved aqueous stability and enhanced sensor performance.

As a particularly significant subclass of MOFs, zeolitic imidazolate frameworks (ZIFs) employ imidazole-based linkers and metal salts, delivering outstanding thermal and chemical resistance. Yang et al. [124] developed a nitrogen-doped ZIF-derived electrode (Z-1000/GCE) via pyrolysis at 1000 °C, resulting in an electrochemical sensor with high electrocatalytic activity toward hydroquinone (HQ) and catechol (CT). The sensor achieved the following:

1. HQ: linear detection range of 1–200 µM with a detection limit of 270 nM;
2. CT: linear range of 1–300 µM with a detection limit of 215 nM.

The enhanced response toward catechol was attributed to increased nitrogen doping and greater porosity, which together facilitated efficient electron transfer and improved analyte interactions. Some other research on electrochemical methods coupled with nanomaterials is described in Table 4.

#### Fire Scene Adaptability of Metal Oxide, Carbon-Based, and Hybrid Nanomaterial Sensors

Table 3 underscores the promising applications of metal oxide nanoparticles (e.g., SnO_2_, ZnO, TiO_2_, NiO) and carbon-based nanomaterials (e.g., CNTs, graphene) in the electrochemical detection of fire-related pollutants. When integrated with electrochemical techniques such as cyclic voltammetry (CV), square-wave voltammetry (SWV), differential pulse voltammetry (DPV), and amperometry, these materials enable the detection of environmentally hazardous compounds like bisphenol A (BPA), nitrophenols, and polycyclic aromatic hydrocarbons (PAHs) at extremely low concentrations, with reported detection limits in the picomolar range.

Their thermal stability, high electrocatalytic activity, and large surface areas ensure reliable signal generation under harsh environmental conditions. In fireground scenarios, these sensors offer several advantages: they are intrinsically resistant to smoke, elevated temperatures, and humidity, and they are compatible with compact, battery-powered systems. Carbon nanostructures further improve sensor performance by enhancing electron transfer and mechanical durability. Selectivity is notably enhanced via π–π stacking interactions, as demonstrated in pyrene-functionalized sensing platforms [131], which facilitate strong binding with aromatic targets such as PAHs.

However, several challenges hinder full deployment in fire scene environments. Most reported sensors are still only validated under aqueous-phase conditions, with limited evidence supporting their use in gas-phase or aerosol detection, which is more relevant in fire settings. Cross-sensitivity, environmental signal drift, and the absence of integrated aerosol sampling interfaces further restrict their real-time, on-site operational viability.

In parallel, metal–organic frameworks (MOFs) and molecularly imprinted polymers (MIPs) have emerged as advanced sensing materials, offering enhanced selectivity and adaptability. MOFs—composed of metal ions or clusters coordinated with organic linkers—possess high porosity, tunable pore architectures, and excellent thermal and chemical stability, which are crucial in the oxidative and thermally unstable environments typical of fire scenes. Notably, zeolitic imidazolate frameworks (ZIFs) and cluster-stabilized MOFs have demonstrated superior stability in aqueous environments. For example, Yang et al. [106] developed a nitrogen-doped ZIF-derived electrode capable of detecting hydroquinone (HQ) and catechol (CT), with linear ranges of 1–200 µM and 1–300 µM and detection limits of 270 nM and 215 nM, respectively.

Similarly, MIPs provide biomimetic molecular recognition via highly selective binding cavities, formed through covalent or non-covalent imprinting strategies. These materials can be combined with electrochemical transduction techniques—such as CV, SWV, and DPV—to detect trace-level combustion byproducts with high fidelity.

Table 4 expands on these capabilities, highlighting nanomaterial-enhanced electrochemical sensors incorporating Cd/Al-LDHS, Fe_3_O_4_-Calix[4]arene, dendritic star co-polymers (Au(G3PPT-co-P3HT)), and graphene-based electrodes. These systems have achieved femtomolar to nanomolar detection limits for analytes such as anthracene, naphthalene, and phenanthrene across various environmental matrices, including tap water, wastewater, cloud water, and oil-contaminated samples.

Techniques like phase-selective AC voltammetry (PSACV) and electrochemical impedance spectroscopy (EIS) further enhance the resolution and analytical accuracy in chemically complex backgrounds. Together, these findings highlight the potential of MOF- and nanocarbon-based electrochemical platforms as highly sensitive, miniaturizable, and fire-resilient technologies for environmental monitoring and firefighter exposure assessment. However, the translation of these systems from bench-scale prototypes to fully integrated, gas-phase-ready sensors remains the key technological challenge for real-world firefighting applications.

### 4.4. Surface-Enhanced Raman Spectroscopy (SERS) Methods for PAH Detection

Conventional Raman scattering is inherently weak, with only approximately 10^−10^ of incident photons contributing to the scattered signal. However, surface-enhanced Raman spectroscopy (SERS) addresses this limitation through the use of noble metal nanostructures, particularly gold or silver, which significantly amplify Raman signals. SERS has shown strong potential for the detection of trace-level PAHs.

For instance, Scanlon et al. [132] employed gold nanofilms to detect PAHs in water, achieving limits of detection (LODs) of 10 ng·L^−1^ for naphthalene and pyrene and 50 ng·L^−1^ for m-triphenyl, with analysis times under 5 min. Mosier et al. [133] further enhanced PAH detection on food-contact surfaces using 1-propanethiol-modified silver nanoparticles, reaching an LOD of 0.27 ng·cm^−2^ for fluoranthene, with good linearity across the tested range. They also embedded DMCX-functionalized silver nanoparticles into sol–gel matrices, enabling the detection of pyrene and naphthalene at concentrations of 3 × 10^−10^ mol·L^−1^ and 1.3 × 10^−8^ mol·L^−1^, respectively. In a different approach, Shen et al. [134] introduced a thin-layer chromatography (TLC)–SERS platform for the direct detection of PAHs in cooking oil, eliminating the need for pre-treatment and achieving LODs as low as 1 ng per spot. Despite these advancements, the inherently weak interactions between PAHs and metal surfaces often limit sensitivity. Functionalization strategies using alkanes, calixarenes, cyclodextrins, and humic substances have been explored to enhance PAH capture. However, many SERS-based detection platforms still achieve only microgram-per-liter (μg/L) detection, which may be insufficient in meeting regulatory or environmental monitoring needs in real-world aqueous systems.

To overcome these limitations, a novel approach integrating surface-enhanced Raman spectroscopy (SERS) with liquid–liquid extraction (LLE) has been developed. This strategy significantly improved the detection limits for the 16 priority PAHs, reducing them to 0.1 μg/L, primarily due to enhanced co-adsorption mediated by iodide (I^−^) ions. Using a rapid and portable SERS technique enhanced with iodide ions, all 16 priority PAHs were successfully detected both qualitatively and quantitatively, with sensitivity ranges spanning 0.1 to 100 μg/L. The addition of LLE further decreased the detection thresholds to the nanogram-per-liter (ng/L) range. This method is simple, low-cost, and environmentally sustainable, while delivering the highest sensitivity reported to date for PAH detection via SERS.

Some other research on electrochemical methods coupled with nanomaterials is described in Table 5.

The incorporation of nanomaterials into fluorescence-based sensors has significantly advanced the field of PAH detection. These nanomaterials serve critical roles as signal transducers, energy donors, or preconcentration substrates for target analytes. As summarized in Table 4, numerous studies have employed nanomaterial-based platforms for the detection of polycyclic aromatic hydrocarbons (PAHs).

The surface functionalization of nanomaterials has enabled the design of highly selective PAH sensors through the integration of molecular recognition elements such as macrocyclic compounds (e.g., cyclodextrins), graphene derivatives, and molecularly imprinted polymers (MIPs). Among various fluorescent nanomaterials, quantum dots (QDs) have emerged as particularly promising tools for PAH sensing due to their unique optical characteristics.

Quantum dots are semiconductor nanocrystals that exhibit exceptional fluorescence properties as a result of quantum confinement effects. Their attributes—including tunable emission wavelengths, broad excitation spectra, and easily modifiable surfaces—make them well suited for the detection of trace levels of PAHs. Functionalized QDs can serve as both signal transducers and molecular recognition elements, particularly when conjugated with target-specific ligands.

Interactions between PAH molecules and QDs may result in either fluorescence quenching or enhancement, which forms the underlying mechanism of many sensitive detection strategies. To improve the selectivity and binding affinity, QDs are often functionalized with macromolecular hosts such as cyclodextrins (CDs) and calixarenes (CAs). Cyclodextrins—composed of α- (six glucose units), β- (seven), or γ- (eight) glucose units arranged in a toroidal configuration—offer hydrophobic cavities capable of selectively encapsulating PAH molecules. When these macrocyclic hosts are covalently attached to QD surfaces, the resulting nanocomposites exhibit enhanced molecular recognition and tailored fluorescence responses, significantly improving both the sensitivity and selectivity in PAH detection platforms.

#### Fire Scene Adaptability of SERS and Fluorescent Nanomaterial Sensors

SERS-based sensors, particularly those enhanced with iodide ions and liquid–liquid extraction (LLE), demonstrate exceptional sensitivity in detecting all 16 priority PAHs, with detection limits down to the ng/L range. These platforms incorporate macrocyclic agents, ligand-functionalized nanoparticles, and magnetic substrates to enhance PAH affinity and allow effective performance in complex aqueous matrices, as summarized in Table 5. Their compatibility with portable Raman spectrometers, rapid response times, and low power consumption make them promising candidates for field deployment.

In fireground conditions, SERS systems benefit from high sensitivity and fast signal acquisition but face limitations including signal reproducibility, substrate degradation, and optical interference from smoke and particulates. Moreover, most reported systems have been validated only in clean aqueous environments, restricting their performance under aerosol-rich and thermally unstable fire scene conditions.

Similarly, fluorescence sensors based on QDs offer high tunability, brightness, and selectivity toward PAHs—particularly when combined with molecularly selective coatings such as MIPs or cyclodextrins. However, challenges including photobleaching, background autofluorescence, and the fragility of optical components limit their practical use in smoky, high-temperature environments.

Both SERS and QD-based fluorescence sensing platforms demonstrate considerable potential but require further engineering and validation for real-time, on-site PAH monitoring in active firefighting environments.

### 4.5. Fluorescence and UV Spectrometry Methods for PAH Detection

**Figure 10:**
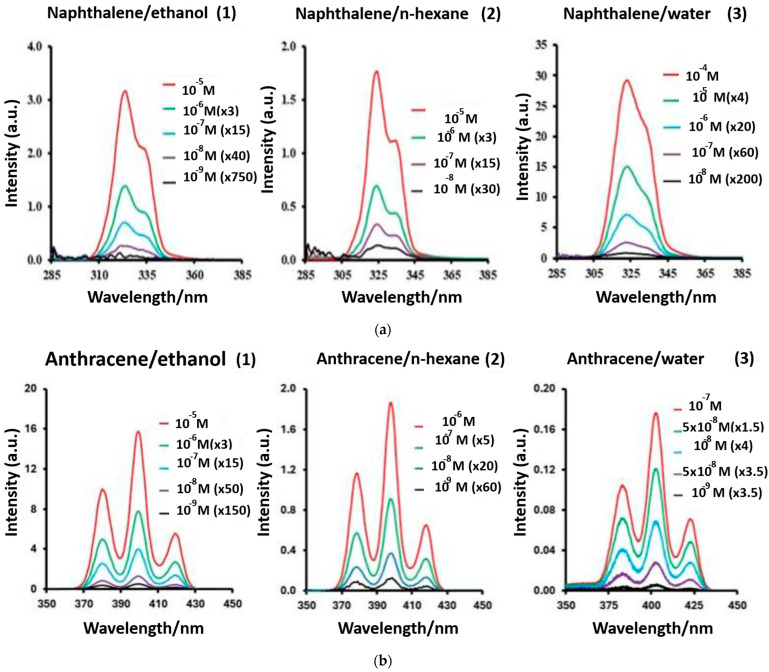
Fluorescence spectra of anthracene and naphthalene. (**a**) Synchronous fluorescence spectra (Δλ = 50 nm) of naphthalene in (1) ethanol, (2) n-hexane, and (3) water. Intensities divided by 10^4^. (**b**) Synchronous fluorescence spectra (Δλ = 44 nm) of anthracene in (1) ethanol, (2) n-hexane, and (3) water. Intensities divided by 10^4^ [152]. Some fluorescence research on PAH detection is described in Table 6.

**Figure 11:**
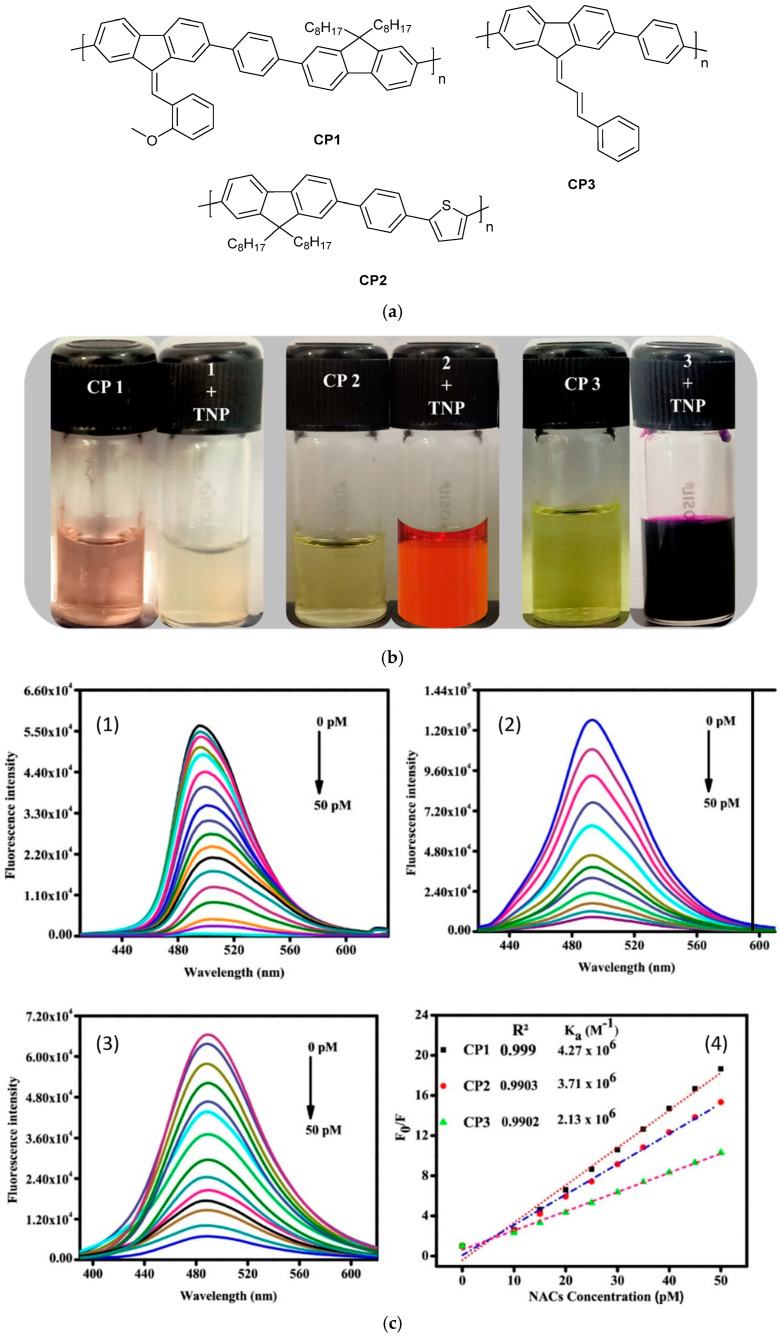
(**a**) Structures of CP1, CP2, and CP3. (**b**) Polymers CP1, CP2, and CP3 (10 μM) changing to different colors with the addition of 500 nM PA in daylight. (**c**) Spectral changes in fluorescence emission behavior of polymers (1) CP1, (2) CP2, and (3) CP3 (10 μM) in aqueous solution (H_2_O/THF (3:2, *v*/*v*)) and (4) changes in the SV plots of polymers CP1, CP2, and CP3 treated with different concentrations of TNP explosive (0–50 pM) [142].

Optical spectrometry—including fluorescence, phosphorescence, and UV–vis techniques—offers sensitive, non-destructive analytical tools for PAH detection in both environmental and industrial samples. These methods are rapid, effective in both gas and liquid phases, and capable of achieving parts-per-trillion (ppt)-level detection. Among them, fluorescence spectroscopy is particularly well suited for PAH analysis due to their rigid conjugated structures, which produce distinct emission spectra under UV excitation, thus enabling multi-component detection (Figure 10). Tropp et al. [151] developed a sensor array composed of six fluorescent conjugated polymers functionalized with 2-phenylbenzimidazole side chains to detect the 16 EPA-priority PAHs via the inner filter effect (IFE). This approach relies on fluorescence quenching rather than specific chemical affinity, and, when combined with principal component analysis (PCA) and linear discriminant analysis (LDA), it achieves high classification accuracy. In comparison to conventional gas chromatography (GC) and high-performance liquid chromatography (HPLC), IFE-based polymer arrays provide rapid, column-free, and low-cost detection, while maintaining an effective balance between sensitivity and selectivity. Taniya et al. [142] reviewed polymer-based fluorescent sensors designed for the detection of nitroaromatic and explosive compounds. These systems—particularly those constructed from biopolymeric or synthetic polymeric materials—are cost-effective, mechanically robust, and capable of producing rapid, visible fluorescence responses through diverse molecular recognition mechanisms (Figure 11).

**Table 6:** Some fluorescence research on PAH detection.

Sensing Technology	Material	Target PAH	LOD	Preparation/Medium	Ref.
**Fluorescence**	Nanomaterial: M-L-cys-CdSeTe/ZnSe/ZnS-GO	Phenanthrene Anthracene	1.07 × 10^−9^ mol/L 1.46 × 10^−9^ mol/L	PAH was in solution form and added to the sensor	[64]
**Fluorescence**	Nanomaterial: M-L-cys-CdSeTeS/ZnS-GO	Phenanthrene	2.26 × 10^−9^ mol/L	PAH was in solution form and added to the sensor	[64]
**Fluorescence**	Nanomaterial: M-SWCNT-QDs	Benzo[a]pyrene Benzo[a]anthracene	-	PAH was in solution form and added to the sensor	[65]
**Fluorescence**	Pyrene-imprinted polythiophene thin film	Pyrene	0.01 × 10^−6^ mol/L	-	[153]
**Fluorescence**	MIP (thymine-based co-polymers)	Benzo[a]pyrene	39.6 × 10^−12^ mol/L	Dimethylsulfoxide (DMSO)	[154]
**Fluorescence**	Fe_3_O_4_-MIPs	Pyrene	9.88 × 10^−8^ mol/L	Water or acetonitrile/water mixture	[155]
**Fluorescence**	Intrinsic fluorescence	Benzo[a]anthracene Chrysene	0.016 µg/L	Dairy products	[59,156]
**Fluorescence**	Intrinsic fluorescence–constant-energy synchronous fluorescence spectroscopy	16 PAHs in air filter	0.058 ng/mL	16 PAHs in atmospheric particulate matter	[157]

### 4.6. Capillary Electrophoresis (CE)

Capillary electrophoresis (CE), also referred to as high-performance capillary electrophoresis (HPCE), is a sophisticated liquid-phase separation technique that utilizes narrow capillaries and a high-voltage direct-current (DC) electric field to drive analyte migration. By combining the principles of electrophoresis and chromatography, CE enables high-resolution separation while requiring only minimal sample volumes in the micro- to nanoliter range. This technique offers several advantages, including rapid analysis, high theoretical plate numbers, cost-effectiveness, and operational simplicity. These attributes make CE a viable and efficient method of analyzing polycyclic aromatic hydrocarbons (PAHs), particularly as an alternative or complement to gas chromatography (GC) in the detection of high-boiling-point PAHs. Several CE variants, such as micellar electrokinetic capillary chromatography (MEKC) and parking capillary chromatography (PCC), have been applied to PAH detection. However, a notable limitation of CE is its relatively low sensitivity when coupled with UV–vis detection, primarily due to the short optical path lengths of the capillaries. Strategies such as multi-pass optical configurations have been proposed to overcome this drawback. Additionally, variability in electroosmotic flow, which is influenced by the sample composition, may compromise method reproducibility.

In a significant advancement, Benhabib et al. [158] developed a portable, microchip-based CE system known as the Mars Organic Analyzer (MOA), capable of detecting PAHs in both laboratory and field environments. The system achieved detection limits ranging from 2000 ppm to 6 ppb, demonstrating substantial potential for in situ environmental analysis, including applications in planetary exploration. Beyond CE, emerging technologies such as nanopore sensing have shown promise for PAH detection, particularly for compounds like benzo[a]pyrene. This method operates by driving macromolecules through nanoscale pores embedded in insulating membranes under pressure. The translocation of analytes through the nanopore temporarily blocks the ionic current, producing signal fluctuations that reflect the molecular size and structural features. In a notable study, Perera et al. [159] utilized β-hemolysin (βHL) nanopores to detect benzo[a]pyrene diol epoxide adducts bound to guanine residues in synthetic oligodeoxynucleotides. Their system generated distinct multi-level current signatures, demonstrating the potential of nanopore technology in monitoring and sequencing mutagenic PAH–DNA adducts. Another promising strategy involves molecular imprinting technology (MIT), which employs synthetic polymers to create analyte-specific binding cavities that mimic natural molecular recognition. These molecularly imprinted polymers (MIPs) offer high selectivity and binding affinity, making them highly suitable for targeted PAH analysis.

### 4.7. NMR

Nuclear magnetic resonance (NMR) spectroscopy is a precise and non-destructive analytical technique that is widely employed to elucidate the structures and compositions of organic molecules. In the context of polycyclic aromatic hydrocarbons (PAHs), NMR is particularly valuable due to the distinct spectral patterns associated with their aromatic ring systems. NMR enables the detection and characterization of both the presence and specific types of aromatic rings in diverse sample matrices, including water, soil, and crude oil. Furthermore, quantitative NMR (qNMR) facilitates the accurate measurement of PAH concentrations. When combined with sample preparation techniques such as solid-phase extraction (SPE), NMR can achieve detection limits in the parts-per-billion (ppb) range or lower, making it a powerful tool for environmental monitoring and pollution assessment. Aromatic hydrocarbons exhibit higher water solubility and significantly greater toxicity to aquatic organisms compared to their aliphatic counterparts. Consequently, in addition to quantifying the total oil content in water, there is a growing need to discriminate between aromatic and aliphatic fractions. While conventional oil-in-water detection techniques—such as laser-induced fluorescence (LIF), ultrasonic acoustics, and optical microscopy—are widely used, they suffer from limitations in sensitivity, selectivity, and the ability to distinguish between dissolved and dispersed hydrocarbons. To overcome these challenges, a hybrid method integrating solid-phase extraction (SPE) with benchtop proton nuclear magnetic resonance (^1^H NMR) spectroscopy, termed SPE-NMR, has been developed. In this approach, reversed-phase SPE is employed to selectively extract and preconcentrate hydrocarbon analytes from the aqueous phase. Subsequent NMR analysis enables the quantitative and selective determination of both aromatic and aliphatic hydrocarbon components. The method utilizes a solvent system consisting of 1% *v*/*v* chloroform in perchloroethylene, which provides an internal reference signal, rendering the system self-calibrating. The SPE-NMR method has demonstrated excellent analytical performance, with detection limits in the 1–100 ppm range, and compares favorably with traditional techniques such as infrared (IR) spectroscopy and gas chromatography (GC). This technique offers a robust, accurate, and cost-effective solution for the routine monitoring of oil contamination in produced water, with the added advantage of directly quantifying aromatic and aliphatic fractions separately [160,161,162,163,164].

### 4.8. Biological Methods

Biosensors have emerged as efficient and versatile tools for the detection of polycyclic aromatic hydrocarbons (PAHs) due to their high selectivity, rapid response, and adaptability to field conditions. Typically, biosensors consist of a biological recognition element—such as enzymes, antibodies, DNA, or microbial cells—combined with a transducer that converts the biorecognition event into a measurable signal [165]. Several types of bioreceptors have been utilized for PAH detection. Enzymatic biosensors employing laccase or tyrosinase have been widely reported, capitalizing on their ability to catalytically oxidize PAHs, thereby generating electrochemical signals. Immunosensors based on monoclonal antibodies have also been developed to detect compounds such as benzo[a]pyrene and phenanthrene with high specificity. DNA aptamer-based sensors have demonstrated the sensitive recognition of PAHs through π–π stacking interactions, enabling both fluorescence and electrochemical detection. Wu et.al. [166] reported the development of an amperometric biosensor utilizing rat cytochrome P450 1A1 (CYP1A1) for the selective and sensitive detection of benzo[a]pyrene (BaP), a well-known carcinogenic PAH. The biosensor was constructed by immobilizing CYP1A1 onto a glassy carbon electrode modified with a Nafion film. Direct electron transfer between the immobilized enzyme and the electrode surface enabled the electrochemical detection of BaP. The biosensor exhibited a linear response to BaP concentrations ranging from 0.1 to 10 μM, with a detection limit of 0.05 μM. Moreover, the sensor demonstrated good stability and reproducibility, indicating its potential for the environmental monitoring of BaP. While biosensors offer exceptional sensitivity and selectivity, their deployment in fireground environments depends on addressing challenges related to sample processing, sensor regeneration, and biological variability. Sample pre-treatment—including filtration, pH adjustment, or solvent extraction—is essential in isolating PAHs and preserving bioreceptor activity, particularly in complex matrices such as smoke, water, or biofluids. The integration of microfluidic systems or solid-phase extraction modules can facilitate automated, field-ready sample processing [167]. Sensor reusability is limited by biofouling and bioreceptor degradation. Approaches such as enzyme immobilization on nanomaterials, electrochemical regeneration, and chemical rinsing have been explored to extend the operational lifespan [168]. For instance, Wu et al. [166] demonstrated a CYP1A1-based biosensor with a detection limit of 0.05 μM for BaP; however, its long-term robustness under fireground conditions remains unvalidated. Biomonitoring applications must also account for individual metabolic differences. Variables such as enzyme expression levels, age, and genetic background influence PAH metabolism, thereby affecting the detection of metabolites versus parent compounds [169]. Consequently, biosensors should be capable of detecting both native PAHs and their metabolites or should be calibrated to specific biological contexts. Addressing these operational challenges is critical in enabling reliable biosensor implementation for firefighter exposure assessment and on-site diagnostics.

### 4.9. Toward Portable Methods

Field-effect transistor (FET) technology offers a promising alternative for the detection of polycyclic aromatic hydrocarbons (PAHs), primarily due to its high sensitivity, rapid response, and compatibility with miniaturized platforms for portable or wearable applications. An illustrative example is the DNA/Cu_2_O-GS-FET sensor, which targets naphthalene and achieves a detection range of 2 × 10^6^ to 3 × 10^7^ nM/L [170]. These sensors operate by transducing interactions between PAH molecules and a chemically functionalized gate surface into measurable shifts in drain current or gate voltage. This electrical readout mechanism enables FET-based platforms to detect trace concentrations in real time with high specificity. Additionally, innovations in materials—such as the integration of graphene, carbon nanotubes, and metal oxides—have improved the selectivity and transconductance of these sensors, making them well suited for PAH detection in complex field environments. Despite these advantages, standard analytical techniques such as GC-MS, HPLC-MS/MS, and CE—although highly sensitive and selective—are poorly suited for deployment in fire stations or field conditions. These systems are bulky, expensive, and require highly trained personnel. Furthermore, they involve time-intensive sample preparation, complex reagent handling, and significant energy and infrastructure requirements. As a result, their use is typically confined to central laboratories, making them ideal for scientific research or retrospective toxicological analysis but impractical for real-time exposure monitoring during or immediately after fire events.

In contrast, electrochemical platforms and FET-based devices have emerged as viable, field-deployable solutions for decentralized PAH detection [171]. Their compact form factors, operational simplicity, and low-cost fabrication make them ideal candidates for on-body exposure monitoring. These technologies address the growing need for immediate hazard assessment in occupational health—particularly for first responders and firefighters, who are routinely exposed to complex mixtures of carcinogenic PAHs and volatile organic compounds (VOCs) during and after fire suppression activities.

For example, a study examining PAH and VOC exposure in firefighters found statistically significant increases in urinary PAH metabolites post-firefighting, especially among personnel involved in attack and search operations [24]. VOCs such as benzene were also elevated in exhaled breath immediately after fire suppression. The strongest correlation for PAH metabolite accumulation was observed approximately three hours post-exposure, highlighting a critical window where real-time monitoring is essential [28]. In this and related work by Fent et al. [12,15,28], samples were analyzed using gold-standard instruments like GC-MS—offering high accuracy but limited to delayed, retrospective analysis. This delay presents a major limitation: firefighting demands time-sensitive decision making that cannot rely on risk assessments delayed by hours or days. Electrochemical and FET-based technologies, by contrast, offer the immediate, on-site evaluation of toxic exposure, enabling prompt intervention and decontamination procedures. These devices have already demonstrated efficacy in detecting airborne pollutants [134,135], urinary biomarkers [136,137], and even analytes in sweat [109,110,111], demonstrating their versatility across diverse biosensing applications. However, despite progress in wearable and portable sensors for other contaminants, no published study has reported a practical, field-deployable sensor for continuous PAH monitoring in firefighting scenarios. Closing this gap could significantly enhance firefighter safety. For instance, a lightweight, integrated FET-based PAH sensor embedded in the outer layer of firefighting gear, as conceptualized in the illustrated design, could continuously monitor airborne PAHs near critical respiratory and dermal zones, alerting users to periods of heightened exposure. Such a system could also inform post-mission decontamination strategies, helping to ensure that personnel are not inadvertently re-exposed to residual toxicants on their gear.

In summary, transitioning from traditional, laboratory-bound instrumentation to real-time, miniaturized FET-based PAH detection systems represents a paradigm shift in occupational exposure monitoring—with the potential to significantly enhance situational awareness, health outcomes, and response protocols for firefighters and other high-risk professions.

**Figure 12:**
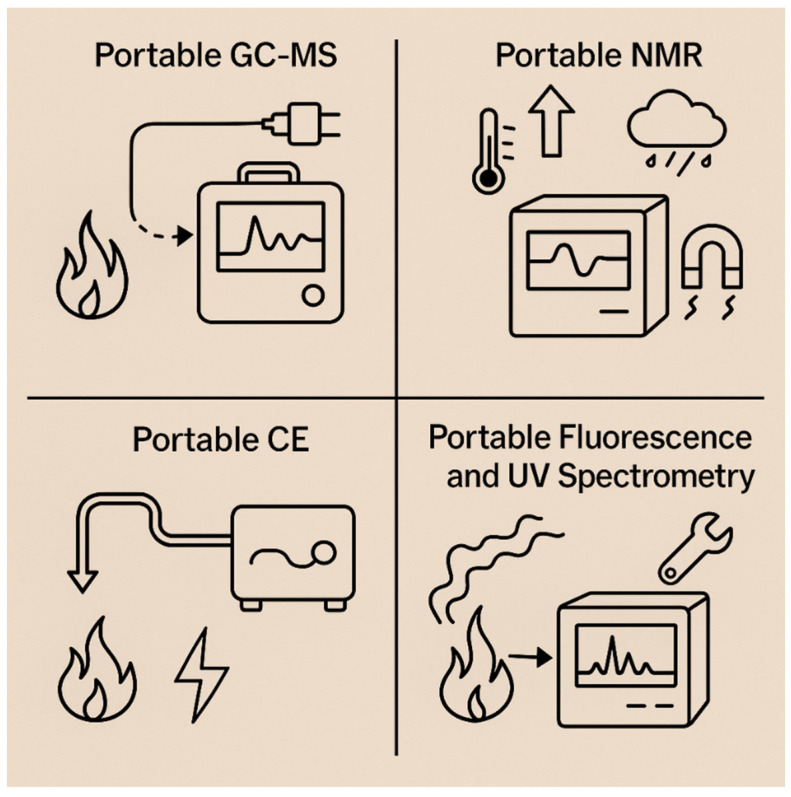
Illustration of portable analytical techniques including GC-MS, NMR, CE, and fluorescence/UV spectroscopy, highlighting their respective operational challenges in firefighting environments, such as thermal instability, electromagnetic interference, power dependence, and optical signal disruption.

To date, significant efforts have been directed toward developing portable versions (Figure 12) of analytical instruments traditionally confined to laboratory use [172,173]. For example, Chen et al. introduced a miniaturized gas chromatograph–mass spectrometer (GC-LIT-MS) integrated with a low-temperature adsorption thermal desorption (LTATD) module based on thermoelectric cooling [172]. This system enables the rapid and sensitive detection of 65 volatile organic compounds (VOCs) within 10 min, achieving a detection limit of 0.12 μg/L for toluene and providing up to a 17-fold improvement in detecting low-boiling-point compounds compared to ambient-temperature adsorption. Weighing 21.7 kg and operating without cryogens, the system demonstrates high reproducibility (RSD < 10%, recovery 91.66–109.12%), indicating promise for field applications such as environmental monitoring and potential fireground deployment. However, challenges remain, including the need for further validation under real-world, high-temperature, and contaminated conditions, as well as concerns about ruggedness, power autonomy, and limitations in achieving laboratory-grade resolutions for complex VOC mixtures without compromising portability. Parallel research has explored the miniaturization of nuclear magnetic resonance (NMR) systems for environmental and water quality applications [174,175]. Despite these technological advances, portable NMR systems face major limitations in firefighting environments. Their sensitivity remains insufficient in detecting trace-level airborne contaminants, such as PAHs and VOCs, which often occur at parts-per-billion concentrations at fire scenes. Environmental instability—especially elevated temperatures, humidity, and electromagnetic interference—can disrupt magnetic field homogeneity and degrade spectral resolutions. Moreover, these systems require carefully prepared liquid-phase samples, making the real-time analysis of airborne gases or aerosols infeasible without complex pre-conditioning. The data acquisition and interpretation process is also time-intensive, requiring trained personnel and sophisticated spectral processing, which undermines their utility for real-time decision making. Lastly, while considered “portable”, current NMR systems lack the ruggedness and power autonomy needed for reliable deployment in mobile, high-risk fireground operations. Collectively, these limitations reduce their viability as frontline diagnostic tools for firefighter exposure or environmental hazard assessment.

Similarly, capillary electrophoresis (CE)—another classical analytical method—has undergone efforts toward portable adaptation for environmental monitoring [176,177]. A notable example includes the optimization of CE via a central composite design for the separation of pharmaceutical contaminants in water [7]. However, multiple constraints hinder its use in fireground scenarios. CE systems are typically optimized for clean aqueous samples in controlled laboratory settings, while fire environments present complex matrices of particulates, gases, and volatile byproducts, often requiring non-standard sample pre-treatment. Additionally, CE depends on precise voltage control, stable temperature conditions, and accurate fluid handling, all of which are difficult to maintain in rugged, unstable field environments. These factors compromise the system’s resolution, reproducibility, and reliability, making CE unsuitable for rapid or in situ analysis during emergency response.

In addition, portable fluorescence and UV spectrometry devices [178,179,180], while capable of the rapid and sensitive detection of various hazardous substances, face substantial limitations in firefighting environments. Fire scenes involve high temperatures, elevated humidity, and dense smoke particulates, all of which can scatter or absorb excitation and emission light, disrupting accurate optical measurements. Such environmental instability impairs spectral fidelity, baseline correction, and calibration reliability. These spectrometers also exhibit limited selectivity in complex smoke matrices, increasing the likelihood of false positives or the misidentification of PAHs and other combustion-related compounds. Furthermore, most portable units require controlled sample presentation, pre-calibration, and careful handling—none of which are feasible during active fire suppression. Additional concerns include the fragility of optical components, dependence on stable power sources, and the need for trained personnel. Collectively, these constraints limit the practicality of using such devices for real-time exposure monitoring or on-site decontamination assessment at fire scenes.

As shown in Table 7, each portable analytical technique presents distinct trade-offs when evaluated for real-time use in fireground environments. Among them, GC-MS offers the highest sensitivity and molecular specificity, capable of detecting VOCs and ignitable liquids at sub-ppb levels within minutes. However, its high cost (often exceeding USD 150,000), reliance on sampling modules (e.g., SPME or thermal desorption), and susceptibility to battery limitations and environmental fragility hinder its sustained deployment in dynamic fire scenarios. Portable NMR systems, although increasingly compact and moderately priced (~USD 100,000), lack the sensitivity required for trace airborne pollutants and require liquid-phase samples. Their spectral fidelity also deteriorates rapidly under high heat, humidity, and electromagnetic interference, making them poorly suited for on-site fire scene analysis. Capillary electrophoresis (CE) performs well in laboratory settings with clean aqueous samples but faces major constraints in the complex, particulate-laden environments typical of firegrounds. Its dependence on a stable voltage, precise fluid control, and high sample purity severely limits its adaptability to field conditions.

In contrast, fluorescence and UV spectrometry devices are notable for their speed and cost-effectiveness. These lightweight, battery-operated systems offer rapid screening capabilities; however, their limited selectivity in complex combustion mixtures, vulnerability to optical interference from smoke and aerosols, and requirement for filtered or clarified sample presentation undermine their accuracy and reliability in operational firefighting settings.

***Phased Technological Roadmap for Deployment of PAH Detection Sensors in Firefighting Applications:*** To ensure the successful deployment of PAH detection technologies in firefighting environments, a phased roadmap is essential, balancing near-term feasibility with long-term innovation (see Table 8). In the short term (1–2 years), efforts should focus on translating validated laboratory platforms—such as electrochemical sensors (e.g., DPV, SWV), iodide-enhanced SERS substrates, and aptamer-based biosensors—into rugged, battery-powered, handheld formats. These devices should be optimized for aqueous-phase sample matrices, such as decontamination rinse water or saliva, and paired with modular sample preparation units (e.g., SPE or microfluidics) to improve field usability. Parallel development should include optical shielding, thermal insulation, and standardization protocols for sensor calibration and baseline correction under dynamic conditions. In the long term (3–7 years), the focus should shift toward aerosol-phase and gas-phase PAH detection using miniaturized SERS or electrochemical arrays integrated with aerosol pre-concentrators or membrane capture systems. This includes embedding sensors into wearable firefighter gear (e.g., helmets, masks, jackets) and enabling wireless data transmission to central monitoring hubs. Additionally, advanced biosensor development should address multi-analyte detection, on-device signal processing with AI-based interference correction, and regenerative biosensing architectures for prolonged field deployment. Long-term strategies should also ensure interoperability with health surveillance systems, enabling real-time biomonitoring aligned with occupational safety standards.

## 5. PAH Removal

Consequently, the removal of PAHs from soil and water sources is strongly recommended. In the atmosphere, PAHs undergo reactions with photochemically generated oxidants, as well as with reactive oxygen and nitrogen species (ROS and RNS), leading to the formation of hydroxylated and nitrated aromatic hydrocarbons. Derivatives such as nitro-, dinitro-, nitro-hydroxy-, and nitro-oxy-aromatic compounds are produced through interactions with ozone and nitrogen oxide radicals [183]. Decontamination techniques can be applied across gas (e.g., air), liquid (e.g., water), and solid (e.g., soil, food) phases. However, removing PAHs from water and air is particularly challenging due to their low solubility and chemical resistance to degradation. Despite these challenges, several treatment methods (such as advanced oxidation processes (AOPs), electrochemical oxidation, and microbial biodegradation) have demonstrated effectiveness. Numerous technologies have been developed to reduce PAH contamination and limit human exposure. Physical processes such as adsorption, filtration, flocculation, sedimentation, and membrane-based separation techniques have successfully reduced PAH concentrations to undetectable levels, although they may encounter issues related to phase transitions and material instability [184,185,186,187,188].

A variety of methods have been developed for the removal of PAHs [189,190,191,192,193,194,195,196,197,198,199,200,201], including physical, chemical, biological, and combined approaches. These include ultrasound treatment, the activated sludge process, coagulation and flocculation, membrane filtration, adsorption, advanced oxidation processes (AOPs), and the Fenton reaction. Table 9 summarizes the different removal techniques for PAHs across various environmental matrices.

This article separately explains each of the methods for the removal of polycyclic aromatic hydrocarbons (PAHs) and the research conducted in this field.

## 6. Conclusions

In conclusion, the rise in urban and wildfires in recent years—largely driven by climate change—has led to a significant increase in environmental PAH levels and direct exposure risks for firefighters. Addressing this issue is critical not only for firefighter health but also for community-wide protection, given the potential for these compounds to spread through air and water systems. Numerous studies have confirmed that firefighters face serious risks from exposure to combustion-derived chemicals, particularly polycyclic aromatic hydrocarbons (PAHs). These findings underscore the urgent need for effective methods to detect and remove hazardous compounds like PAHs from firefighter environments. Recent advances in PAH analysis using chromatographic techniques such as GC, HPLC, and SFC—as well as electrochemical and SERS-based optical sensors—have been extensively reviewed. There is a clear need to replace traditional sample preparation methods with advanced extraction and purification techniques to enhance PAH recovery from both liquid and solid samples. Although most GC methods rely on flame ionization detection (FID), studies using the more sensitive MS/MS detectors remain limited. Precise optimization of the column type and length, stationary phase, and film thickness, as well as temperature programming, is essential to improve the resolution and reduce the analysis time in GC workflows. Similarly, while HPLC methods often utilize fluorescence detection, the adoption of advanced MS/MS detectors is still insufficient. Challenges also persist in UPLC-MS/MS methods, particularly with short columns and small particle sizes, which can impact robustness. Emerging SFC techniques have shown promise in analyzing water samples, but their application to solid and complex liquid matrices remains inadequate. In contrast, electrochemical and optical sensing methods are gaining traction due to their enhanced sensitivity through nanomaterials, low cost, reduced analysis time, and potential for miniaturization and in situ use. Electrochemical sensors can achieve high sensitivity via the synergistic effects of nanomaterials on catalytic activity and surface modifications of working electrodes. Many electrochemical sensors reviewed in this work have demonstrated detection limits below regulatory thresholds and have been successfully applied to real-world samples including wastewater, river water, tap water, and rain water. Additionally, optical methods based on SERS, employing nanomaterials, have achieved significant Raman signal enhancements, leading to high sensitivity. However, their application has largely been limited to the detection of a narrow range of PAHs, highlighting the need for multi-analyte sensor development. Many existing sensing techniques are confined to liquid-phase analysis, which limits their utility in broader monitoring applications. Other persistent challenges include electrode fouling, peak overlap due to interfering compounds, and over-voltage effects that suppress electron transfer in electrochemical sensors. For SERS-based sensors, signal degradation due to continuous laser irradiation and distortion caused by functionalized molecules must also be addressed.

## Figures and Tables

**Table 1:** Comparative analysis of REMPI-eMIMS and GC-MS for naphthalene and phenanthrene in stored and freshly prepared samples.

Sample Condition *	Compound	Theoretical (µg/L)	GC-MS (µg/L)	REMPI-eMIMS (µg/L)
**Stored—Sample 1**	Naphthalene	2.91	3.8	3.9
**Stored—Sample 2**	Naphthalene	5.82	4.6	4.8
**Fresh—Rep 1**	Naphthalene	5.82	4.7	5.1
**Stored—Sample 1**	Phenanthrene	2.37	1.3	1.4
**Stored—Sample 2**	Phenanthrene	4.74	1.5	1.6
**Fresh—Rep 1**	Phenanthrene	4.74	1.7	1.9

* Samples included four PAHs (naphthalene, acenaphthene, fluorene, phenanthrene), prepared in 2 L water volumes and analyzed by both methods to assess REMPI-eMIMS accuracy relative to GC-MS.

**Table 2:** Applications of metal particles in sensor detection.

Pollutant	Sensing Material	Technique	LOD (nM)	Detection Range	Ref.
**Naphthalene**	2D BP:Ti_3_C_2_Tx	LSV	1.6	0.02–40 µM	[101]
**BPA**	PtNPs/Ti_3_C_2_Tx	CV	32	50 nM–5 µM	[102]
**4-NP**	Nb2CTx/Zn–Co–NC	DPV	70	1–500 µM	[103]
**4-NP**	Cu–curcumin	DPV	68.2	0.1–1030 µM	[104]
**HQ**	MWCNTs–Ti_3_C_2_	DPV	6.6	2–150 µM	[105]
**CT**	N–Co–Fe–HCS	DPV	75	0.5–500 µM	[106]
**HQ**	CT	DPV	80	0.5–1500 µM	
**Naproxen**	Ni–Co LDHs	DPV	2	0.01–435 µM	[107]

**Table 3:** **Applications of metal oxide nanoparticles in electrochemical sensor detection.** LOD = Limit of Detection; CV = Cyclic Voltammetry; SWV = Square Wave Voltammetry; DPV = Differential Pulse Voltammetry; ITO = Indium Tin Oxide; CNT = Carbon Nanotube.

Pollutant	Sensing Material	Technique	LOD (nM)	Detection Range	Ref.
**BPA**	Tyrs-rGO/Mn_3_O_4_/ITO	CV	10	0.05–100 µM	[110]
**PHE**	GCE–PANI–NiO	CV	0.732 (pM)	7.6–14 µM	[111]
**2-NP**	ZnO/RuO_2_	SWV	52.2 (pM)	0.1 nM–0.01 mM	[112]
**Catechin**	Pv/MnO_2_/f-MWCNT/GCE	SWV	2	2–950 µM	[113]
**BPA**	ZnO/CNT/IL	SWV	9	0.002–700 µM	[114]
**BPA**	TiO_2_/AuNTAS	Amperometry	6.2	100 nM–38.9 µM	[115]
**CT**	Ag–TiO_2_ electrode	Amperometry	24.9	1–15 µM	[116]
**ANT**	ARS–SBA15/GCE	DPV	0.5 (pM)	1 pM–10 nM	[117]
**Acetaminophen**	CuO–Gr/CPE	DPV	8	0.025–5.3 µM	[118]
**Eugenol**	CoO/ZnO/GCE	DPV	4	0.049–179.8 µM	[119]

**Table 4:** Electrochemical detection of PAHs using nanomaterials.

Sensor	Detection Method	PAH Analyte	LOD	Matrix	Ref.
**Cd/Al-LDHS/GCE**	DPV	Anthracene	0.5 × 10^−15^ mol/L	Electrolyte solution	[125]
**Fe_3_O_4_–Calix[4]arene @ CdSe**	SWV	Anthracene	0.11 × 10^−6^ mol/L	Tap water	[67]
**Fe_3_O_4_–Calix[4]arene @ CdSe**	SWV	Naphthalene	4.29 × 10^−6^ mol/L	Tap water	[67]
**ARS-SBA15/GCE**	DPV	Anthracene	0.5 × 10^−12^ mol/L	Wastewater	[117]
**AQS/PDDA/ITO**	CV	Phenanthrene	0.50 × 10^−12^ mol/L	Cloud water	[126]
**AQS/PDDA/ITO**	CV	Phenanthrene	-	Rain water	[126]
**Au(G3PPT-co-P3HT)**	PSACV	Anthracene	2.62 × 10^−9^ mol/L	Oil-polluted wastewater	[127]
**Au(G3PPT-co-P3HT)**	ACV	Phenanthrene	1.42 × 10^−9^ mol/L	Oil-polluted wastewater	[127]
**Au(G3PPT-co-P3HT)**	CV	Phenanthrene	12.62 × 10^−9^ mol/L	Tap water	[128]
**PAA/GO/SPCE**	SWV	Anthracene	6.7 × 10^−7^ mol/L	Electrolyte solution	[129]
**GO/SPCE**	SWV	-	7.42 × 10^−7^ mol/L	Electrolyte solution	[128,130]
**ITO/PAA films**	LSV	Anthracene	3.79 × 10^−6^ mol/L	Electrolyte solution	[130]
**Carbon-rich monolayer on ITO**	EIS	Pyrene	-	Water	[80]

AQS/PDDA/ITO = anthraquinone sulfonate/poly(diallyldimethylammonium chloride)/indium–tin oxide; ARS-SBA15/GCE = alizarin red S-functionalized mesoporous silica material SBA15 on glassy carbon electrode; Au(G3PPT-co-P3HT) = dendritic star co-polymer, generation 3 poly(propylene thiophene) (G3PPT)-co-poly(3-hexylthiophene) (P3HT) star co-polymer on gold electrode; Cd/Al-LDHS/GCE = cadmium/aluminum layered double hydroxides on a glassy carbon electrode; CV = cyclic voltammetry; DPV = differential pulse voltammetry; EIS = electrochemical impedance spectroscopy; GO/SPCE = graphene oxide—screen-printed carbon electrode; PAA/GO/SPCE = polyamic acid–graphene oxide–screen-printed carbon electrode; PSACV = phase-selective alternating-current voltammetry; SWV = square-wave voltammetry; LSV = linear sweep voltammetry; ITO = indium–tin oxide; ACV = alternating-current voltammetry.

**Table 5:** SERS sensors for PAHs.

Affinity Agent Type	Sensor/Substrate	Analyte	LOD	Matrix	Ref.
**Macromolecule**	b-CD dimer@Ag@SiO_2_ NPs	Perylene	0.1 × 10^−6^ mol/L	DCM	[135]
**Macromolecule**	b-CD-AgNPs	Anthracene	10 × 10^−6^ mol/L	Water	[136]
		Pyrene	7.5 × 10^−6^ mol/L		
**Macromolecule**	b-CD-SH-AuNPs/PGMA-b-CD	Pyrene	0.8 × 10^−9^ mol/L	-	[137]
		Anthracene	2.4 × 10^−9^ mol/L		
**Macromolecule**	GNPS-DSNB	Benzo[a]pyrene	2 × 10^−9^ mol/L	Sea water	[138]
**Macromolecule**	AuNP–alginate gel network	Benzo[a]pyrene	0.485 × 10^−9^ mol/L	River, spring, tap water	[139]
**Macromolecule**	AuNPs-DMCX	Pyrene	0.5 × 10^−9^ mol/L	Artificial sea water	[140]
**Macromolecule**	AuNPs-DMCX	Anthracene	0.5 × 10^−9^ mol/L	Artificial sea water	[140]
**Polymers**	AuNPs-GMA-EDMA	Anthracene	0.93 × 10^−7^ mol/L	Water	[141]
**Polymers**	AuNPs-GMA-EDMA	Phenanthrene	4.5 × 10^−7^ mol/L	Water	[141]
		Pyrene	1.1 × 10^−7^ mol/L		
**Polymers**	pNIPAM-coated nanostars	Pyrene	-	Gas phase	[142]
**Polymers**	AgNO_3_-PVP dendrites	Fluoranthene	0.45 × 10^−9^ mol/L	-	[143]
**Polymers**	IP6-AuNPs	Benzo[a]pyrene	1 mg/L	EtOH	[144]
**Polymers**	Hydroxylamine-reduced AgNPs	Naphthalene	1 × 10^−12^ mol/L	Water	[145]
		Phenanthrene	0.1 × 10^−9^ mol/L		
**Ligands**	Citrate–AuNPs	Benzo[a]pyrene	0.5 × 10^−6^ mol/L	River water	[146]
		Benzo[g,h,i]perylene	0.25 × 10^−6^ mol/L		
**Ligands**	AgNPs—LG	Anthracene	1 × 10^−6^ mol/L	-	[147]
		Benzo[c]phenanthrene	1 × 10^−7^ mol/L		
**Ligands**	GNS-DS-C_10_H_21_	Benzo[a]pyrene	0.1 × 10^−6^ mol/L	Water–MeOH	[148]
		Fluoranthene	0.32 × 10^−6^ mol/L		
		Naphthalene	31 × 10^−6^ mol/L		
**Ligands**	HAs-AgNPs	Fluoranthene	1.3 × 10^−7^ mol/L	Acetone	[149]
		3,4-Benzopyrene	1.3 × 10^−7^ mol/L		
**Magnetic NPs**	Fe_3_O_4_@Ag Fe_3_O_4_@AuNR assemblies Fe_3_O_4_@Au core–satellite MNPs	Perylene	0.8 × 10^−6^ mol/L	-	[150]
		Benzo[a]pyrene	0.8 × 10^−6^ mol/L		
		Pyrene	1 × 10^−6^ mol/L		
		Anthracene	5 × 10^−6^ mol/L		
		Phenanthrene	20 × 10^−6^ mol/L		

**Table 7:** Fire scene technical adaptability.

Technique	Cost (USD)	Response Time	Environmental Robustness	Accuracy/Sensitivity	Sample Preparation	Ref.
**Portable GC-MS**	150,000–300,000	~10 min	Moderate (field-usable but limited high-temperature resilience)	High sensitivity (sub-ppb), lab-grade separation	Requires solid/liquid/vapor sampling (e.g., SPME, probes)	[169]
**Portable NMR**	~100,000	Tens of minutes to hours	Low (sensitive to EMI, temperature, humidity)	Low sensitivity for airborne ppb-level pollutants	Requires liquid samples; not suited for gas-phase analysis	[170]
**Portable CE**	~50,000–100,000 (estimated)	Minutes to tens of minutes	Low (not stable in rugged environments)	Moderate in clean aqueous matrices; poor for complex smoke mixtures	Requires precise injection; clean aqueous samples; capillary conditioning	[181]
**Fluorescence/UV**	Few thousands–tens of thousands	Seconds to a few minutes	Low–moderate (optical interference; some ruggedized models exist)	Moderate (ppm–ppb in clear matrices); low specificity in smoky air	Minimal preparation; requires filtered/clear presentation to reduce light scattering	[182]

**Table 8:** Phased technological roadmap for fireground PAH detection.

Phase	Strategy
**Short-Term (1–2 years)**	Translate validated lab sensors (electrochemical, SERS, aptamer-based) into handheld field devices
	Optimize sensors for aqueous-phase samples (e.g., rinse water, saliva)
	Integrate modular sample preparation units (e.g., SPE, microfluidics)
	Develop environmental protection features (optical shielding, thermal insulation, calibration protocols)
**Long-Term (3–7 years)**	Develop gas-phase PAH sensors with aerosol pre-concentration and capture membranes
	Embed sensors into wearable firefighter gear (helmets, jackets, masks)
	Enable wireless data transmission to centralized monitoring systems
	Advance biosensors for multi-analyte detection and regenerative capabilities
	Integrate with occupational health surveillance platforms for real-time exposure tracking

**Table 9:** Various methods for removal of polycyclic aromatic hydrocarbons in different matrices.

PAH	Method	Efficiency of Removal	Sample	Ref.
Naphthalene	UV–Vis	62%	Sea water	[189]
Pyrene and benzo[a]pyrene	Adsorption	40% and 48%	Synthetic wastewater	[190]
16 PAHs	Photocatalyst ozonation and UV	57%	Offshore-produced water	[187]
Naphthalene, phenanthrene, anthracene	Bioremediation	100%, 95.4%, 73.8%	Oilfield-produced water	[191]
Naphthalene and acenaphthene	Adsorption	100% to 97%	Water treatment plant	[192]
Anthracene, phenanthrene	Fenton process	85.47%, 63.16%	Textile dying sludge	[193]
16 PAHs	Phytoremediation	89%	Wastewater	[194]
16 PAHs	Biodegradation	67.27%	River	[195]
Pyrene, benzo[a]pyrene	Magnetic floatation	89.9%, 66.9%	Sea water	[196]
Phenanthrene, naphthalene	Oxidation	90.1%, 97.5%	Soil	[188]
Naphthalene, phenanthrene	Air-assisted liquid–liquid microextraction	82.0% to 116.6%	Water	[185]
Phenanthrene	Adsorption	90%	Wastewater	[197]
Naphthalene and fluorene	Oxidation adsorption	92% to 100%	Produced water	[198]
Pyrene, fluoranthene, chrysene	Precipitation method	99%, 98%, 87%	Marine sediments	[186]
Naphthalene, anthracene	Oxidation	97%, 95%	Landfill leachate	[199]
16 PAHs	Electrochemical advanced oxidation	99.9%	Petroleum-contaminated water	[200]

## Data Availability

No data are available for this study.
